# *CoverageTool*: A semi-automated graphic software: applications for plant phenotyping

**DOI:** 10.1186/s13007-019-0472-2

**Published:** 2019-08-06

**Authors:** Lianne Merchuk-Ovnat, Zev Ovnat, Orit Amir-Segev, Yaarit Kutsher, Yehoshua Saranga, Moshe Reuveni

**Affiliations:** 1 Institute of Plant Sciences, Agricultural Research Organization (ARO), The Volcani Center, P. O. Box 6, 5025001 Bet Dagan, Israel; 2 Hamacabim 4, Sderot, Israel; 3 The Robert H. Smith Institute of Plant Sciences and Genetics in Agriculture, The Hebrew University of Jerusalem, Rehovot, Israel

**Keywords:** Phenotyping, Image analysis, Senescence, Tissue culture, Ground coverage, Root and leaf projected surface area, YCbCr, RGB, DPI

## Abstract

**Background:**

Characterization and quantification of visual plant traits is often limited to the use of tools and software that were developed to address a specific context, making them unsuitable for other applications. *CoverageTool* is flexible multi-purpose software capable of area calculation in cm^2^, as well as coverage area in percentages, suitable for a wide range of applications.

**Results:**

Here we present a novel, semi-automated and robust tool for detailed characterization of visual plant traits. We demonstrate and discuss the application of this tool to quantify a broad spectrum of plant phenotypes/traits such as: tissue culture parameters, ground surface covered by annual plant canopy, root and leaf projected surface area, and leaf senescence area ratio. The *CoverageTool* software provides easy to use functions to analyze images. While use of *CoverageTool* involves subjective operator color selections, applying them uniformly to full sets of samples makes it possible to provide quantitative comparison between test subjects.

**Conclusion:**

The tool is simple and straightforward, yet suitable for the quantification of biological and environmental effects on a wide variety of visual plant traits. This tool has been very useful in quantifying different plant phenotypes in several recently published studies, and may be useful for many applications.

**Electronic supplementary material:**

The online version of this article (10.1186/s13007-019-0472-2) contains supplementary material, which is available to authorized users.

## Background

Phenotyping solutions that allow fast yet accurate and informative measurement of traits of interest are means to facilitate linking phenotypic and molecular data [1]. Acquiring reliable and accurate phenotypic data with high throughput remains a challenge [1, 2]. Numerous phenotyping platforms have been developed for various traits with differences in automation and throughput levels [3–6], yet, there is continued need to develop further solutions [7, 8].

*CoverageTool* is simple and straightforward software, yet it can be applied for the quantification of a wide variety of biological and environmental effects that yield a visual phenotype. This tool, initially developed for these specific objectives, has been very useful in quantifying such effects [9, 11, 12]. It has also been applied to the biological question of senescence rates in the analyses of multiple sets of plant shoot photos [10]. *CoverageTool* is currently used to quantify tissue culture parameters.

*CoverageTool* image analysis is based on two color spaces: RGB (red, green & blue) or alternatively YCbCr (where Y is the luminance or brightness and Cb & Cr are color components). These color spaces are used as metrics to assess how close similar shades of color are one to another. The tool enables the ‘selection’ of desired areas in an image and ‘ignoring’ of undesired ones. With uniform image acquisition conditions or their standardization, it is possible to uniformly analyze images with high-throughput followed by a suitable statistical test. For scanned images *CoverageTool* can measure areas (e.g. cm^2^) based on the dots per inch (DPI) information embedded in the image file (which is the case for scanned images). For photographed images, coverage can be calculated in percentages—as DPI has no meaning in this context. The area percentages can later be rescaled appropriately to cm^2^ if the image contains an item of known dimensions.

As we found *CoverageTool* to be very useful, it has been made available as freeware for the benefit of plant researchers as well as other potential users. In this paper we demonstrate the potential uses of this tool, including its detailed application in published research papers and provide a step-by-step user guide.

## Implementation

*CoverageTool* (‘Coverage.exe’) is an interactive graphical WIN32 application written in C++ (Visual Studio 2008 Express Edition). There are no LINUX or IOS versions.

The application accepts 24-bit BMPs as input and allows assessment of foreground coverage selected by up to 10 different shades, while other up to 10 different shades can be designated background and ignored. The application displays the original image—and an image in which the background has been erased—side-by-side. The various foreground and background shades are defined by mouse-clicking the shades in the original image. The area of the foreground is calculated assuming that the BMP has correct embedded DPI information (which when converting a JPEG to BMP is usually the case).

*CoverageTool* enables the calculation of the percentage of coverage as well as area in cm^2^. There are some considerations that have to be taken into account while acquiring the images, and in some cases it is necessary to pre-process the images before using the *CoverageTool* as described below.

### Image set acquisition and pre-processing

In all cases, open your image using ***MSPaint***, and then save it as a 24-bit BMP file.

**Fig. 1:**
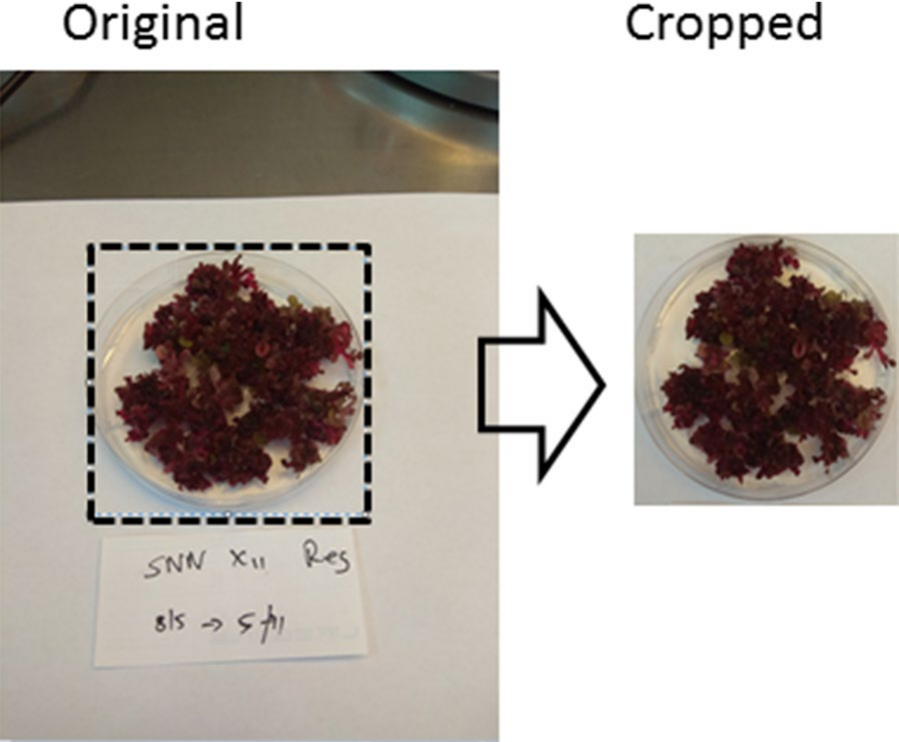
An example for image preparation for analysis by using the ‘crop’ function in *MSPaint*

For relative area coverage calculated as percentage, the uniformity of the set image acquisition is highly important. When capturing images, the same settings must be applied throughout, especially the distance from the object. Otherwise, it would be necessary to include in the image an object of known dimensions, which will enable the standardization of the dataset during pre-processing. For example, a petri dish borderline was used as common scale and cropped in accordance using ***MSPaint***’s ‘crop’ function (Fig. 1).

It is possible to include number of separate samples on the same sheet (each time erasing all but one and analyzing it). *CoverageTool* ‘Coverage.exe’ software and instructions for use-are found in Additional files 1, 2, 3 and 4.

**Fig. 2:**
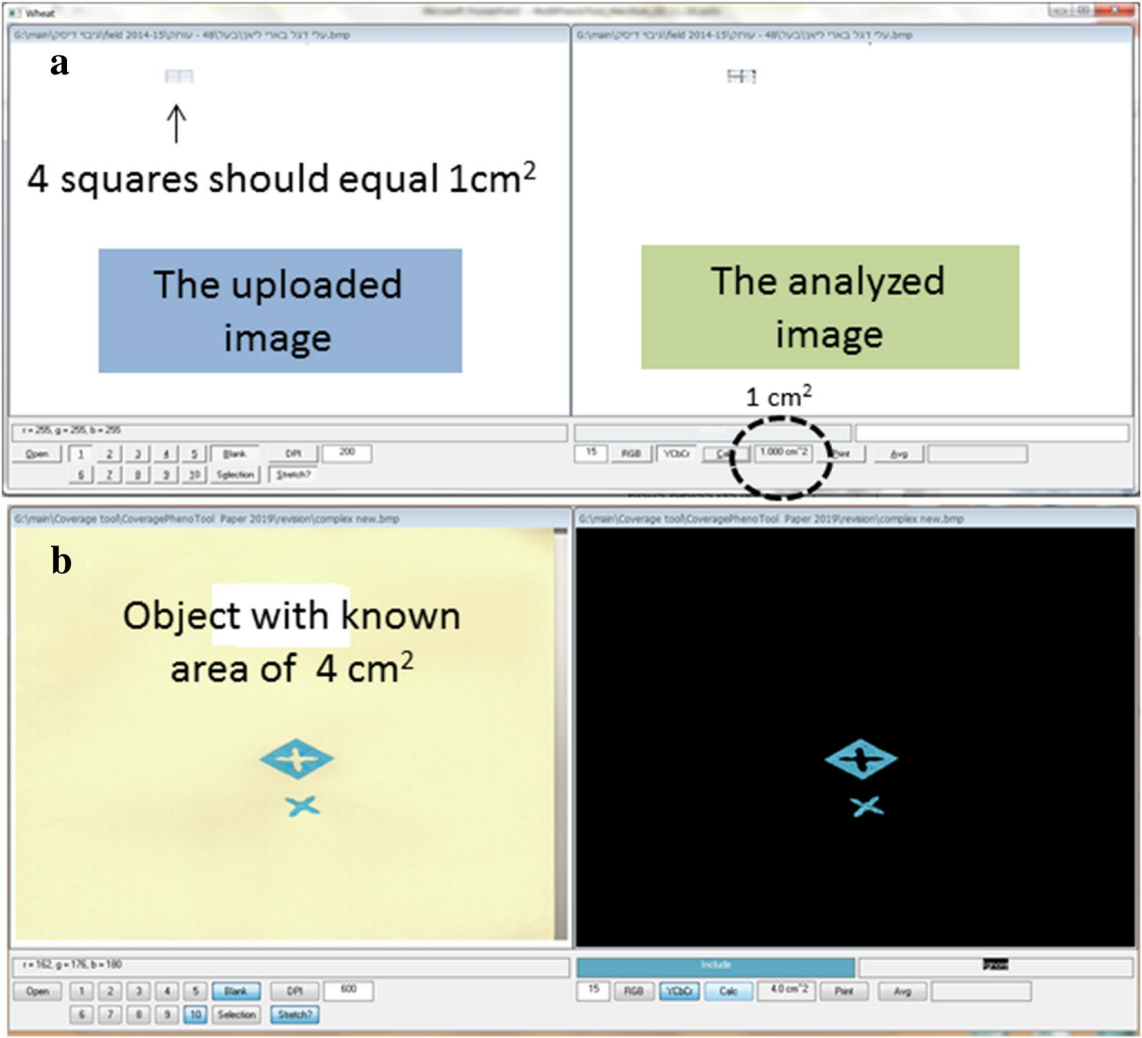
**a** Verification of *CoverageTool*’s area calculation capability. **b** Verification of *CoverageTool*’s area calculation capability of a more complexed object

Quality control: To demonstrate *CoverageTool*’s ability to correctly calculate areas, an ‘A4 square-pattern’ sheet where 4 squares equals 1 cm^2^, was scanned. All but a 2 × 2 array of 0.5 cm squares was erased (Fig. 2a) and the white background was selected to be ‘ignored’. Finally we sampled the colors of the squared pattern paper to account as ‘coverage’ and calculated the area, which was indeed calculated by *CoverageTool* as equal to 1 cm^2^. Figure 2b shows calibration for a more complexed object of a known area of 4 cm^2^.

### Color selection

Any image to analyze contains three parts.

Foreground: the items the relative coverage of which needs to be calculated, e.g. (green) canopy.

Background: the area not covered by canopy, e.g. bare soil.

Area to ignore: patches of the image which obscure/do not contain either foreground or background, (foreign objects) e.g. sample label or netting.

*CoverageTool* provides the user with the ability to define custom color datasets by “sampling” pixel colors from the image to cover the range of colors in the measured object area. Pixels ‘close’ to one of the specified colors according to a RGB or YCbCr metric (within user-specified tolerance) are considered part of the measured object, while pixels ‘further’ away are considered as background. In the same manner the user may sample pixels to ignore, which are not part of the measured object and not part of the background—but are present in the image.

In order to achieve the best representation of the dataset, the process of color sampling should be applied for a number of images, representing the extremities of the entire dataset (each time improving the selection and saving it). Once color selection has been optimized, it can be later applied to the entire dataset in a uniform manner.

Changing the tolerance level determines the ‘closeness’ of pixels in the image to one of the colors specified in the custom dataset according to an RGB or YCbCr metric and has a dramatic effect on the analysis. The same tolerance level must be applied to the entire set to ensure uniformity.

### Calculations

Let N be the number of pixels in the image, let C be the number of pixels that were near to one of the selected colors to include, and let I be the number of pixels that were near to one of the selected colors to ignore.

The coverage percent (Foreground) is: (C × 100)/(N − I)

If B is the number of background pixels, then B = N − C − I

Thus the coverage percent (Foreground) is also: (C × 100)/(C + B)

The foreground area in cm^2^ is: C/(DPI/2.54) ^2^

The RGB or YCbCr color comparison metric selected must be applied uniformly across the entire dataset. Note that RGB requires higher color comparison tolerances. The YCbCr method deemphasizes the brightness of the pixel and concentrates on its hue.

The RGB algorithm assesses the similarity of colors using the differences of the RGB components of the pixel in the picture (rgb1)—and the reference pixel mouse-clicked (rgb2):

$$\left( {{\text{r1 }} - {\text{ r2}}} \right)^{{\text{2}}} + {\text{ }}\left( {{\text{g1 }} - {\text{ g2}}} \right)^{{\text{2}}} + {\text{ }}\left( {{\text{b1 }} - {\text{ b2}}} \right)^{{\text{2}}} < {\text{ tolerance}}^{{\text{2}}}$$

In the YCbCr algorithm, RGB values are converted to YCbCr (Y is the luminance or brightness and Cb and Cr are color components) and the similarity of colors is assessed by:

$$\left( {{\text{Cb1 }} - {\text{ Cb2}}} \right)^{{\text{2}}} + {\text{ }}\left( {{\text{Cr1 }} - {\text{ Cr2}}} \right)^{{\text{2}}} + {\text{ }}\left( {{\text{Y1 }} - {\text{ Y2}}} \right)^{{\text{2}}} /{\text{ 9 }} < {\text{ tolerance}}^{{\text{2}}}$$

The same tolerance is applied to all selection channels.

### Instructions

Instructions to use *CoverageTool* can be found also in Additional files 2 and 3. After preparation of the dataset as described, run the program ‘Coverage.exe’. *CoverageTool* (Additional file 1).

## Results

In order to demonstrate the applicability of *CoverageTool* we provide a range of case studies where it was used to characterize plant physiological and tissue culture phenotypes [9–12].

### Case study 1—Projected leaf area

A full analysis of a flag leaf blade Projected Surface Area (PSA) dataset, which demonstrates the analysis process including decision making is documented in Additional file 4. This dataset comprises of scanned images of two flag leaf blades collected from each experimental plot of a field experiment aimed to characterize a Teff (*Eragrostis tef (Zucc) Trotter*) collection which comprises of six genotypes.

- As presented in Additional file 4, first, a basic 4-color ‘Selection’ was defined based upon the first image to be analyzed. A calculation of PSA under the default tolerance of 35 was performed. Inspection of the results obtained in terms of coverage was then made and tolerance was increased to 40 for better coverage. This adjustment ensured that the unselected black pixels were taken into account in the calculation.
- The next image was then analyzed using the initial selection. However, after application of the selection the leaves were not fully covered. A fifth color was added to the selection and seemed sufficient at that stage.
- Analysis of the third image necessitated the addition of two red colors to the selection.
- The fourth image was analyzed successfully indicating that the selection was ready to be saved for use (inclusion of 7 colors).
- All images were processed under this selection and tolerance. Coverage initially displays images in its image window by stretching or compressing them into the window area. In this case, the pixel-dimension of the images is much larger than the pixel-dimension of Coverage’s image window. It’s therefore, necessary to inspect various areas of the image by zooming into relevant areas viewed in ‘Stretch’ed mode. Clicking ‘Stretch’ results in a zoomed view with 1 screen pixel per image pixel for optimal resolution.

**Fig. 3:**
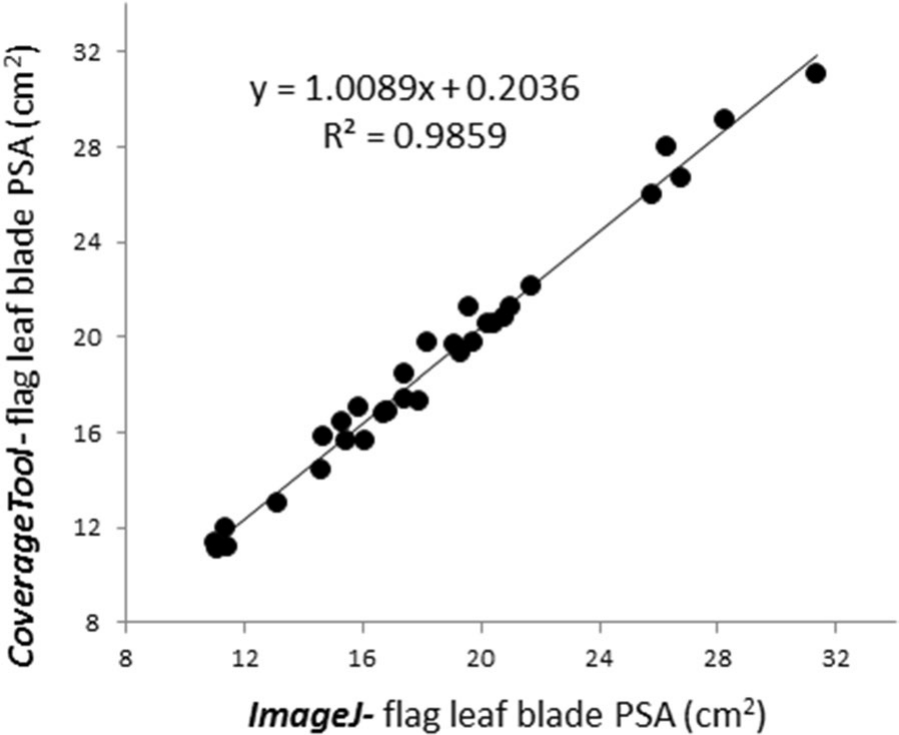
Correlation between results obtained for Teff flag leaf bade projected surface area (cm^2^) using *CoverageTool* (Y axis) and *ImageJ* (X axis) in cm^2^

For validation, the same dataset was analyzed using the *ImageJ* color default thresholding algorithm. The results obtained from both tools are presented in Table 1. A highly significant correlation (p < 0.0001) was found between the results obtained for Teff flag leaf PSA using *CoverageTool* and *ImageJ* (Fig. 3)

**Table 1:** Means StDv of Teff flag leaf blade projected area (cm^2^) analyzed by *Coverage Tool* as well as with *ImageJ* color thresholding default algorithm

Plot #	Image #	Genotype	2 FL area cm^2^	FL area cm^2^	Mean	Std error	2 FL area cm^2^	FL area cm^2^	Mean	Std error
			*CoverageTool*				*ImageJ*			
7	20	35-1	19.40	9.70			19.33	9.67		
10	10	35-1	15.90	7.95			15.85	7.92		
16	28	35-1	22.20	11.10			21.67	10.83		
24	23	35-1	29.20	14.60	10.84	1.42	28.27	14.14	10.64	1.38
2	11	35-2	19.70	9.85			19.63	9.82		
15	2	35-2	19.40	9.70			21.23	10.61		
20	30	35-2	20.60	10.30	9.95	1.64	20.24	10.12	10.18	1.59
30	26	35-3	20.90	10.45			20.83	10.41		
5	25	35-3	11.20	5.60			11.16	5.58		
13	22	35-3	31.20	15.60			31.09	15.55		
21	6	35-3	19.80	9.90			19.73	9.87		
23	24	35-3	20.60	10.30	10.37	1.27	20.53	10.26	10.33	1.23
1	12	44A-361	11.40	5.70			11.36	5.68		
3	21	44A-361	15.70	7.85			15.41	7.71		
14	3	44A-361	13.10	6.55			13.13	6.57		
25	5	44A-361	26.10	13.05	8.29	1.42	26.01	13.01	8.24	1.38
9	8	44B-361	17.10	8.55			17.04	8.52		
11	13	44B-361	16.90	8.45			16.84	8.42		
18	29	44B-361	28.10	14.05			26.28	13.14		
27	16	44B-361	26.80	13.40	11.11	1.42	26.18	13.09	10.79	1.38
4	14	Cont Teff	11.10	5.55			11.06	5.53		
6	27	Cont Teff	16.80	8.40			16.74	8.37		
12	9	Cont Teff	18.00	9.00			18.44	9.22		
17	55	Cont Teff	12.00	6.00			11.39	5.69		
19	15	Cont Teff	21.70	10.85	7.96	1.27	21.23	10.61	7.89	1.23

**Fig. 4:**
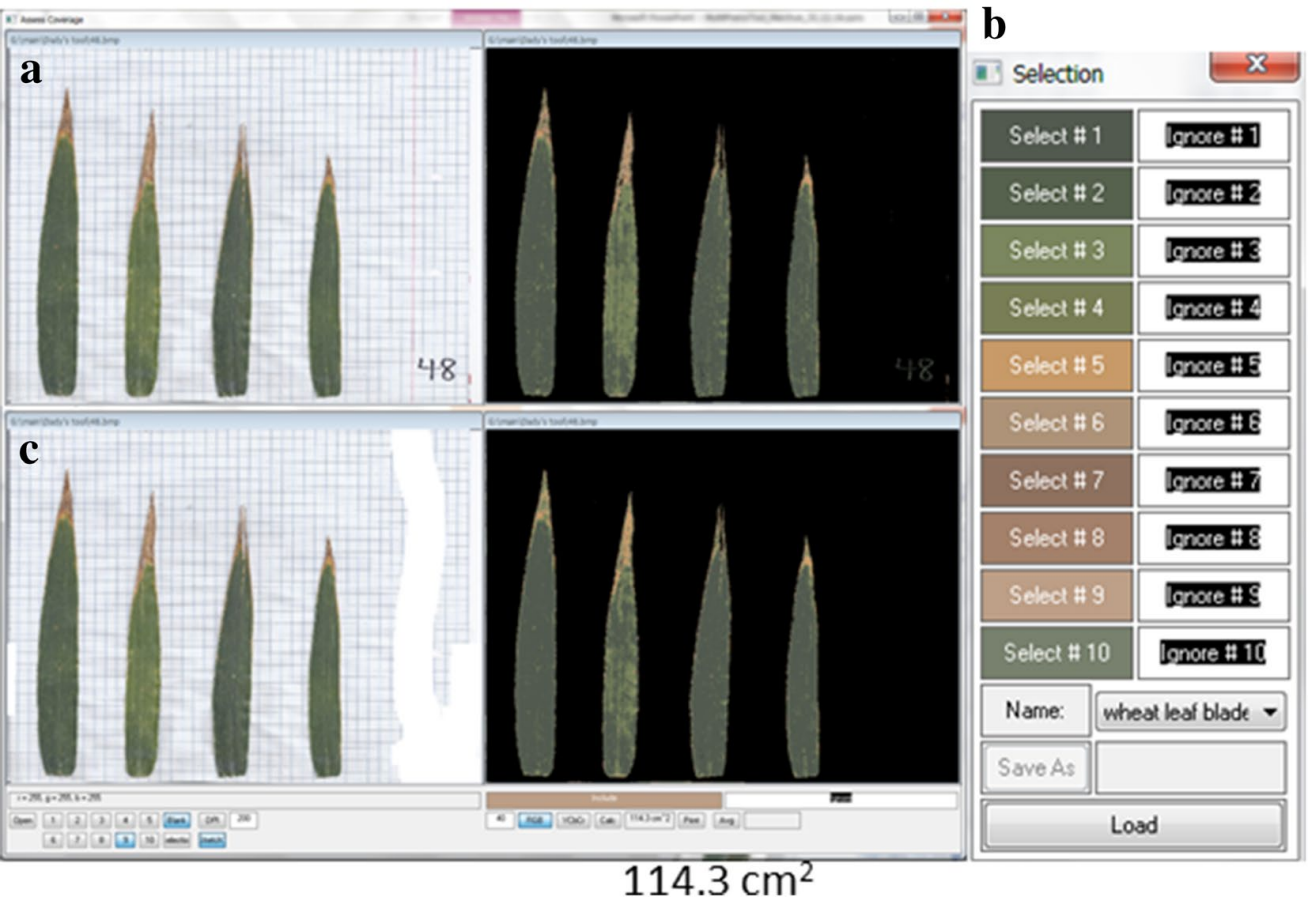
**a** Wheat leaf blades projected surface area calculation: four flag leaves scanned on an A4 sheet of paper. Colors to represent leaves were sampled to be taken into account, with tolerance level of 40. **b** Same as (**a**)—after blanking out of artifacts. **c** 10-channel ‘Selection’ specification dialog box

Another example for flag leaf blade PSA analysis by *CoverageTool* is presented [Fig. 7 and Table 3 in 11] as part of a drought experiment, which included a bread wheat cultivar and its near introgression line. For the measurement of this phenotype ([11]), four flag leaf blades for each experimental plot were sampled and mounted on an A4 sheet of paper and scanned as a 24-bit BMP file (Additional file 5, Fig. 4a). In this case, since we were analyzing leaf area—and not relative coverage—only leaf colors to include (Fig. 4b) needed to be set (there was no need to specify colors to ignore). Next, the brush was used to ‘blank’ out the thin red margin line, the color of which resembles shades in the dry leaf ends (Fig. 4c).

### Case study 2—Root projected surface area

Scanned wheat root images (two genotypes, two water availability treatments, three sampling dates, five soil layers and five replicates) were analyzed to determine root PSA using *CoverageTool* (Figs. 1, 3 in [9]). The experimental setting is detailed in Merchuk-Ovnat et al. 2017. Here is a brief description of the procedure detailed in the article.

**Fig. 5:**
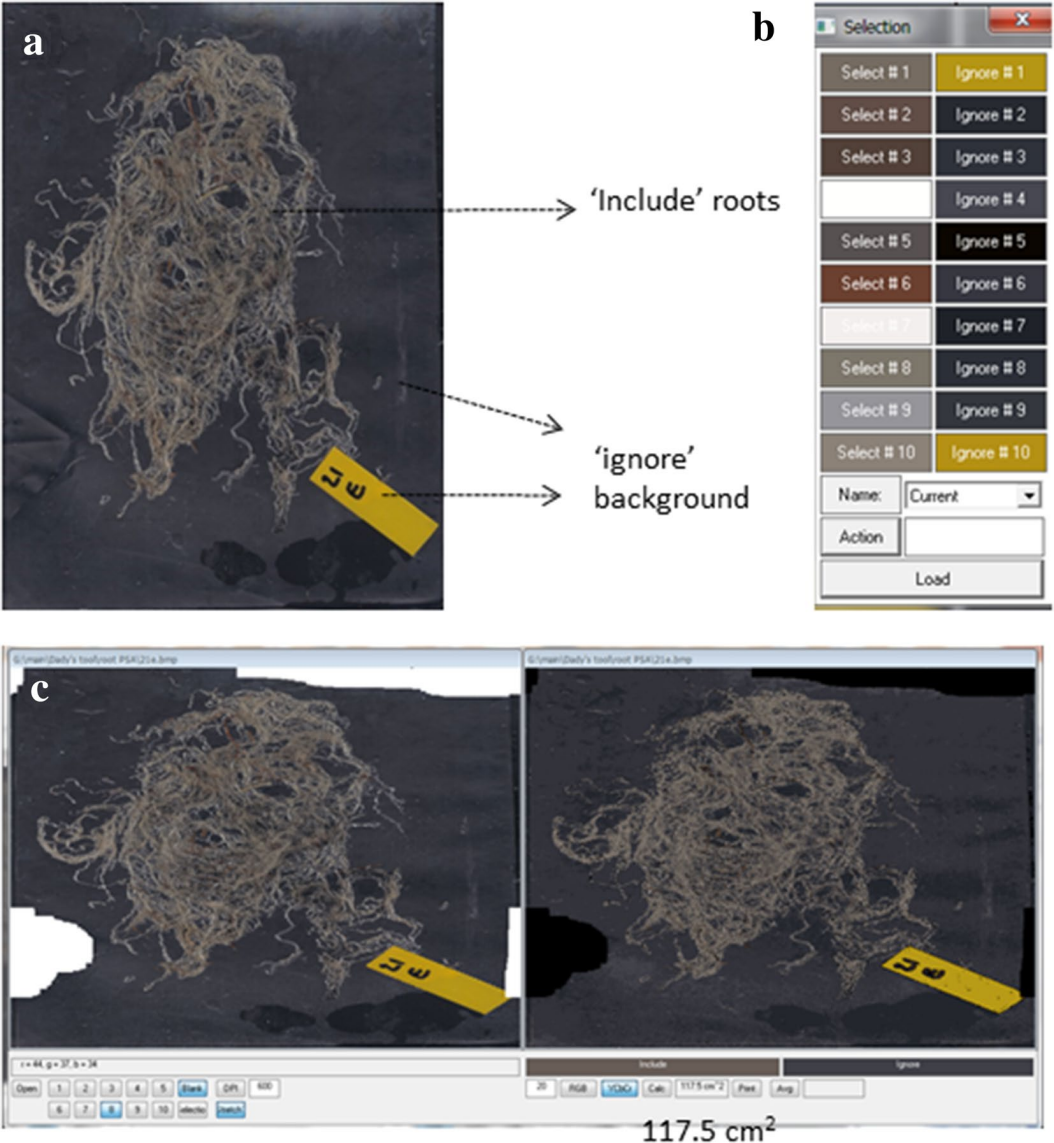
**a** Wheat roots spread and scanned on an A4 sheet of paper with a black background. **b** Selection made to include and ignore, made by sampling in *CoverageTool.* **c** *CoverageTool* Root projected surface area calculation: an example for pre-processing done using the ‘Blank’ feature to erase background noise

**Fig. 6:**
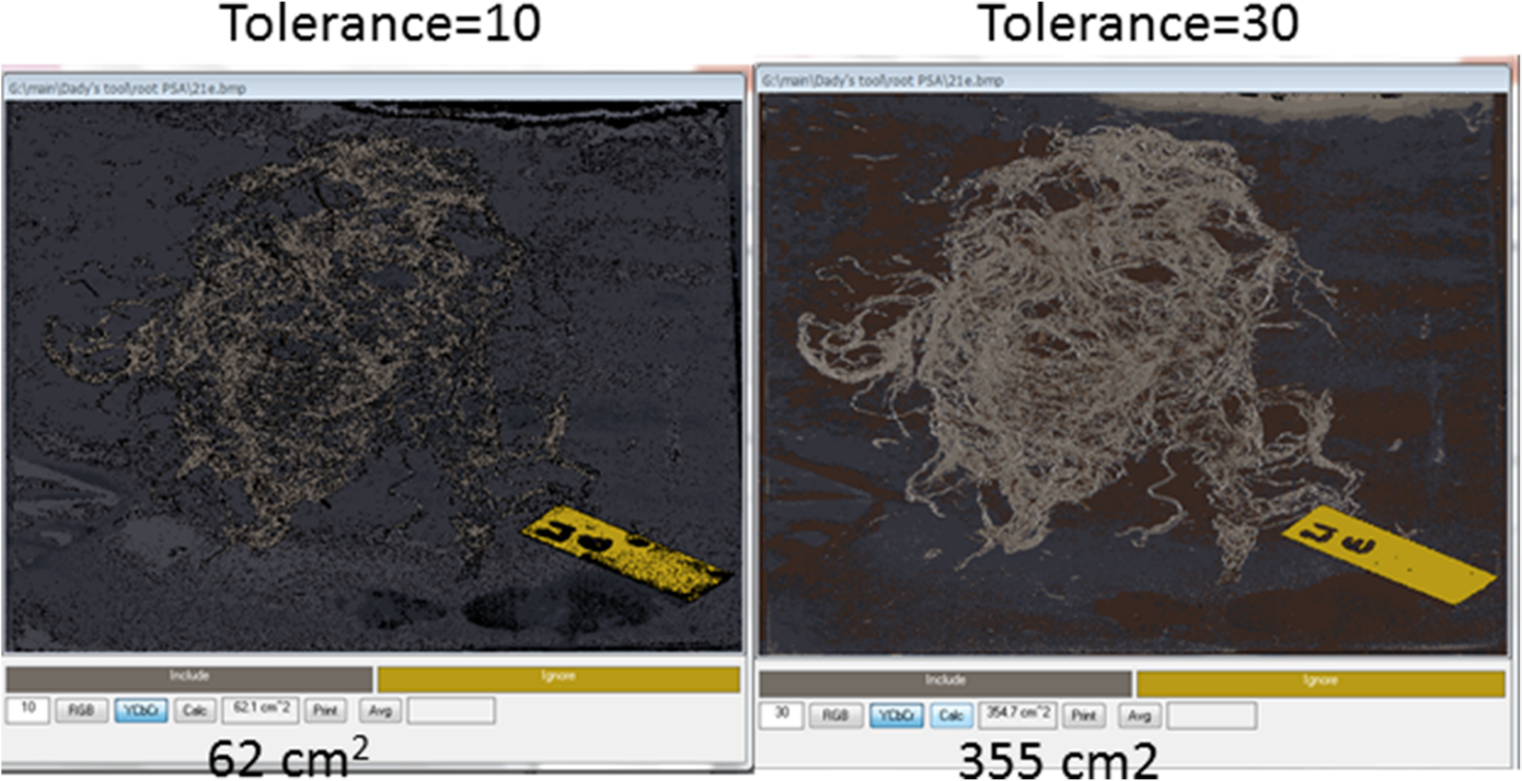
Tolerance level adjustment: different results obtained using too low (left, 10) or too high (right, 30) levels

- Roots were washed, spread over the scanner’s glass top with a black background (Fig. 5a).
- Images were scanned and saved as a 24-bit BMP files (Additional file 6) and opened using *CoverageTool.*
- Ten shades covering the range of root colors were visually sampled from the root images (Additional file 2), as well as shades which do not represent roots to ignore (background Fig. 5b). Pixels ‘close’ to one of the specified colors according to a RGB metric (within user-specified tolerance) were considered part of the measured object, while pixels further away were considered background.
- This process was repeated for several root samples of the same experimental set in order to achieve the best representation of the dataset. Color selection was saved as ‘root area wheat’ and was used to analyze the entire set under the same tolerance level.
- After calculation of the root area, pieces of background which were included as roots were erased (Fig. 5c, Additional file 2. section 13) as their colors were very similar to those of the roots. *CoverageTool* enables erasing of artifacts using the ‘Blank’ feature.
- Changing the tolerance level (in the text box to the left of the ‘RGB’ button) controls the ‘closeness’ of pixels to one of the specified colors according to a RGB or YCbCr metric and had a dramatic effect on the analysis. Figure 6 illustrates the different results obtained using a too low (left, 10) or too high (right, 30) tolerance level, as opposed to the better fit of Fig. 5c, 20. Any error in separation of roots from background is done uniformly across the entire dataset, allowing for quantitative comparison of area ratios from different images in the dataset. This approach was used to analyze 300 scanned images (60 sand tubes, five layers each) and to test the calculated results statistically [9].

### Case study 3—Leaf senescence rate

**Fig. 7:**
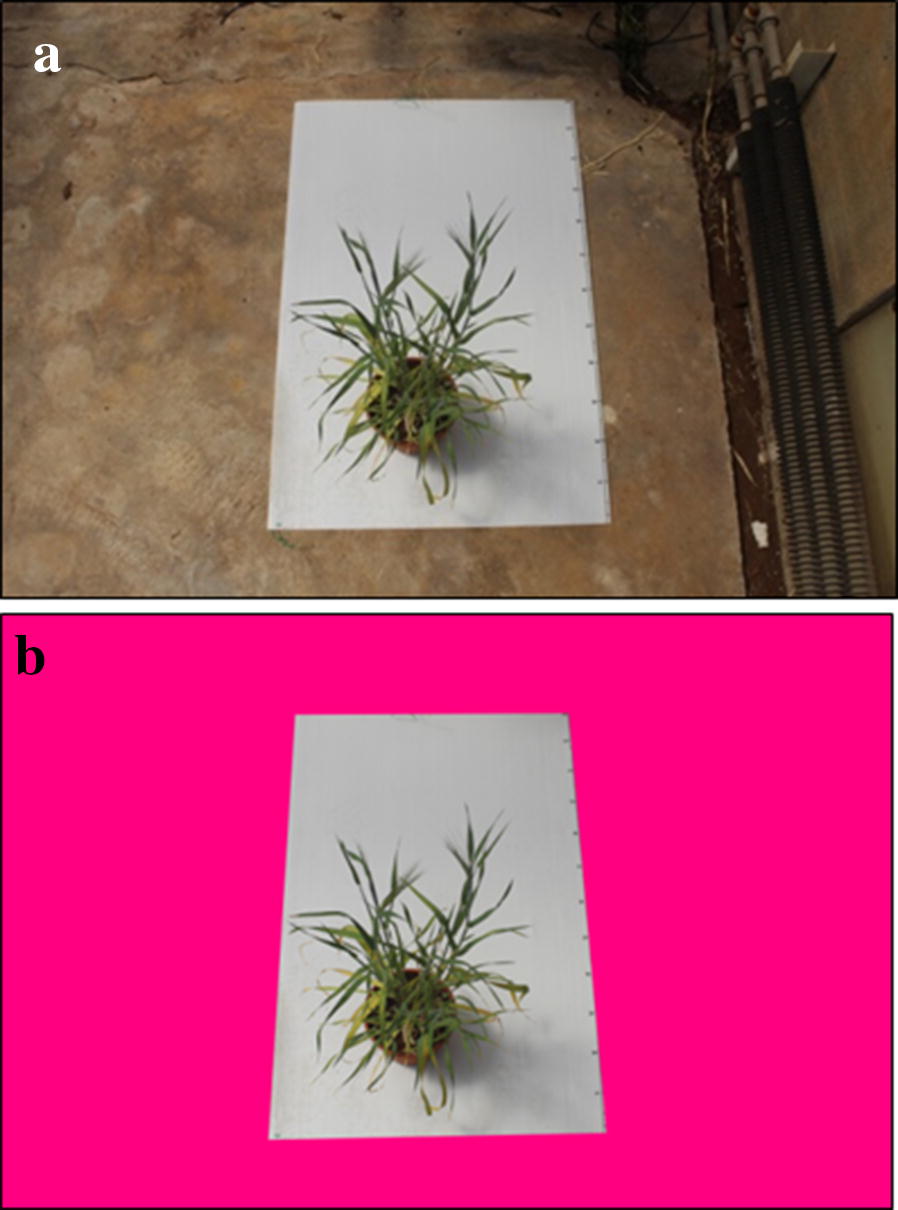
**a** Senescence rate calculation in barley pot-experiment: photos taken at 66 and 78 days after planting, on a background of a standard (80 × 120 cm) white sheet. **b** Pre-processing was performed on the entire dataset, to remove background having shades of color very similar to those of the plants. The image area not including the standard white sheet was excluded by replacing it with a non-plant color (pink)

**Fig. 8:**
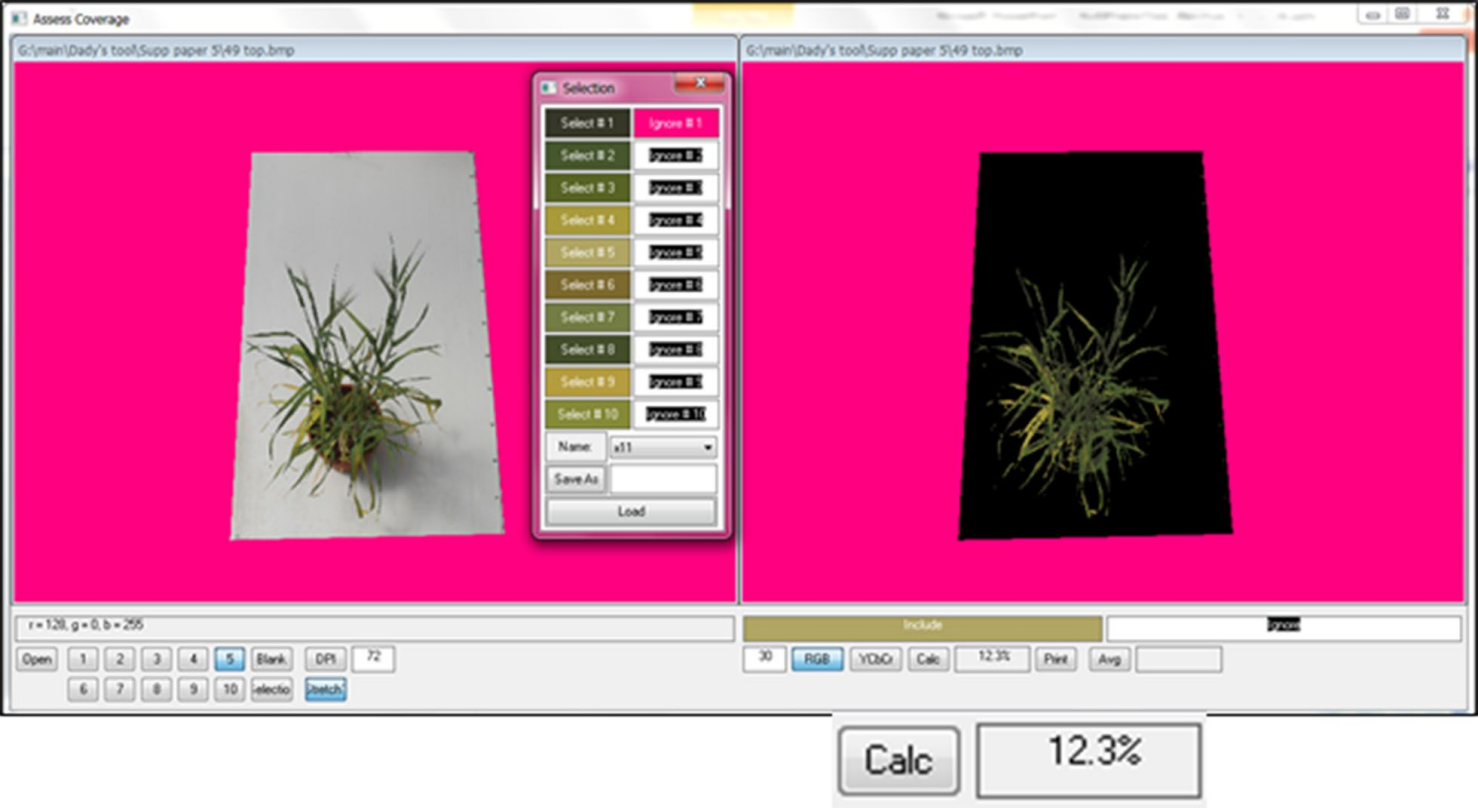
All greens and yellow-browns were sampled to be taken into account, while the pink background was sampled to ignore using a tolerance level of 30, calculating %

For the analysis of leaf senescence in a barley drought pot-experiment [10], we used two sets of photos taken with a Canon EOS1200 camera at 66 and 78 days after planting, against a background of a standard (80 × 120 cm) white sheet (Fig. 7a, b, Additional file 7). *CoverageTool* was used to calculate the ratio of yellow/brown areas to the total leaf area (greens and yellow-browns, Fig. 8). Senescence was calculated for each plant as the increase (∆) of yellow/brown to the total between 66 and 78 days after planting [10].

In this case study, pre-processing was performed on the entire dataset, to remove background having shades of color very similar to those of the plants. The image area not including the standard white sheet was excluded by replacing it with a non-plant color (pink, Fig. 7b), throughout the entire dataset (~ 200 images). Then, the ratio of the percentage of yellow-browns characterizing the senescing areas to the projection area of the entire plant was calculated (see [10]).

### Case study 4—Early canopy ground cover

**Fig. 9:**
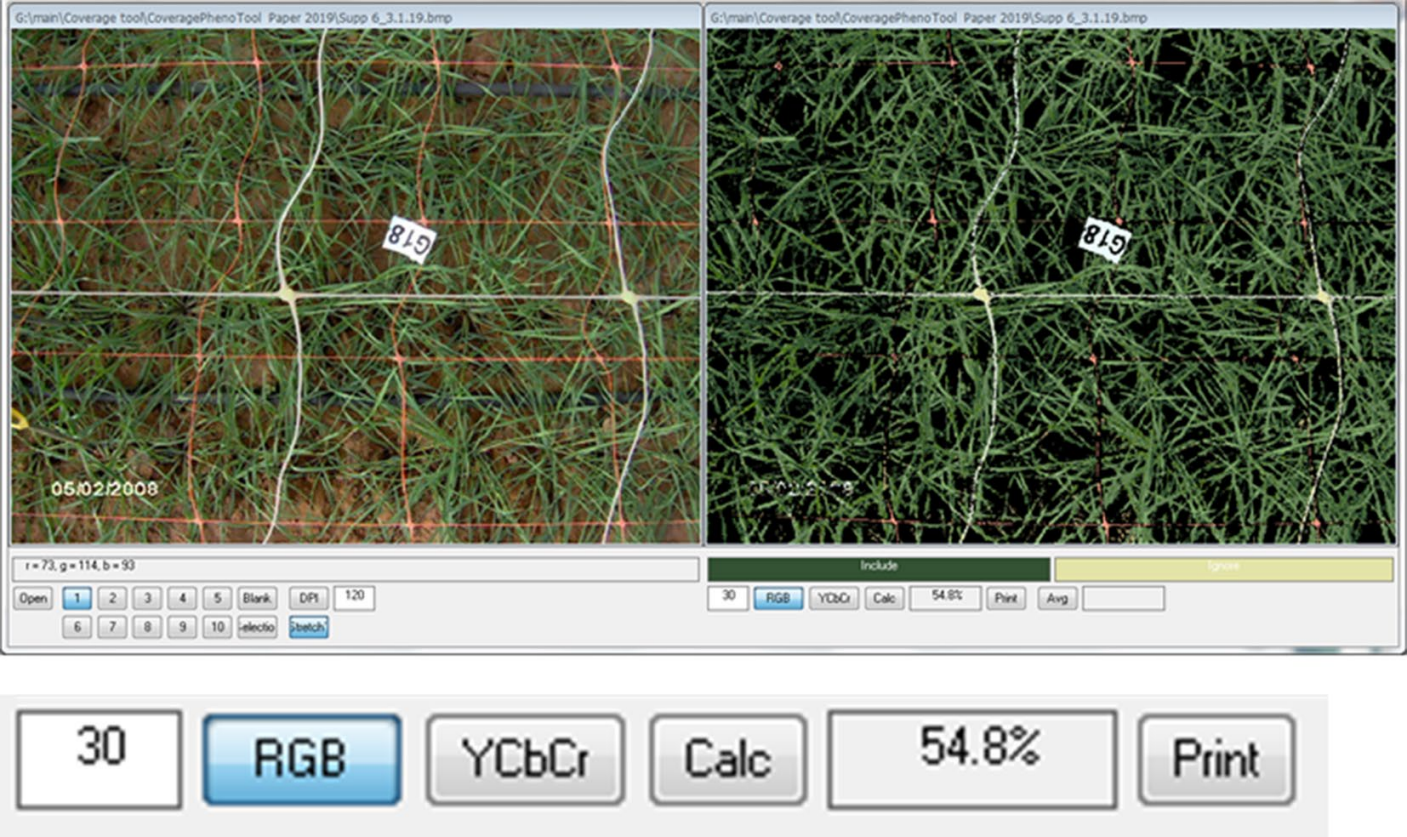
Early ground cover in wheat: an example of a photos taken from above the plots; colors were sampled to be taken into account as canopy while the ‘white and orange’ netting and ‘black and white’ plot’s numbering were selected to ignore. Tolerance level of 30 and was used to calculate the ‘percentage of ground cover by canopy’

The first use of *CoverageTool* was part of the effort to characterize the drought response in wheat recombinant inbred lines *(T. turgidum* ssp. *dicoccoides x T. turgidum durum)* [11]. *CoverageTool* was created in order to quantify ground coverage by canopy in % for each plot. A set of photos was taken vertically from above the plots under the same settings (height of camera from the ground, light, same camera, see Additional file 8). Then, the canopy colors were sampled to be taken into account (foreground), whereas the bare soil brown shades were not selected (background, Fig. 9). White and orange netting and black & white plot-numbering were selected to be ‘Ignored’.

**Fig. 10:**
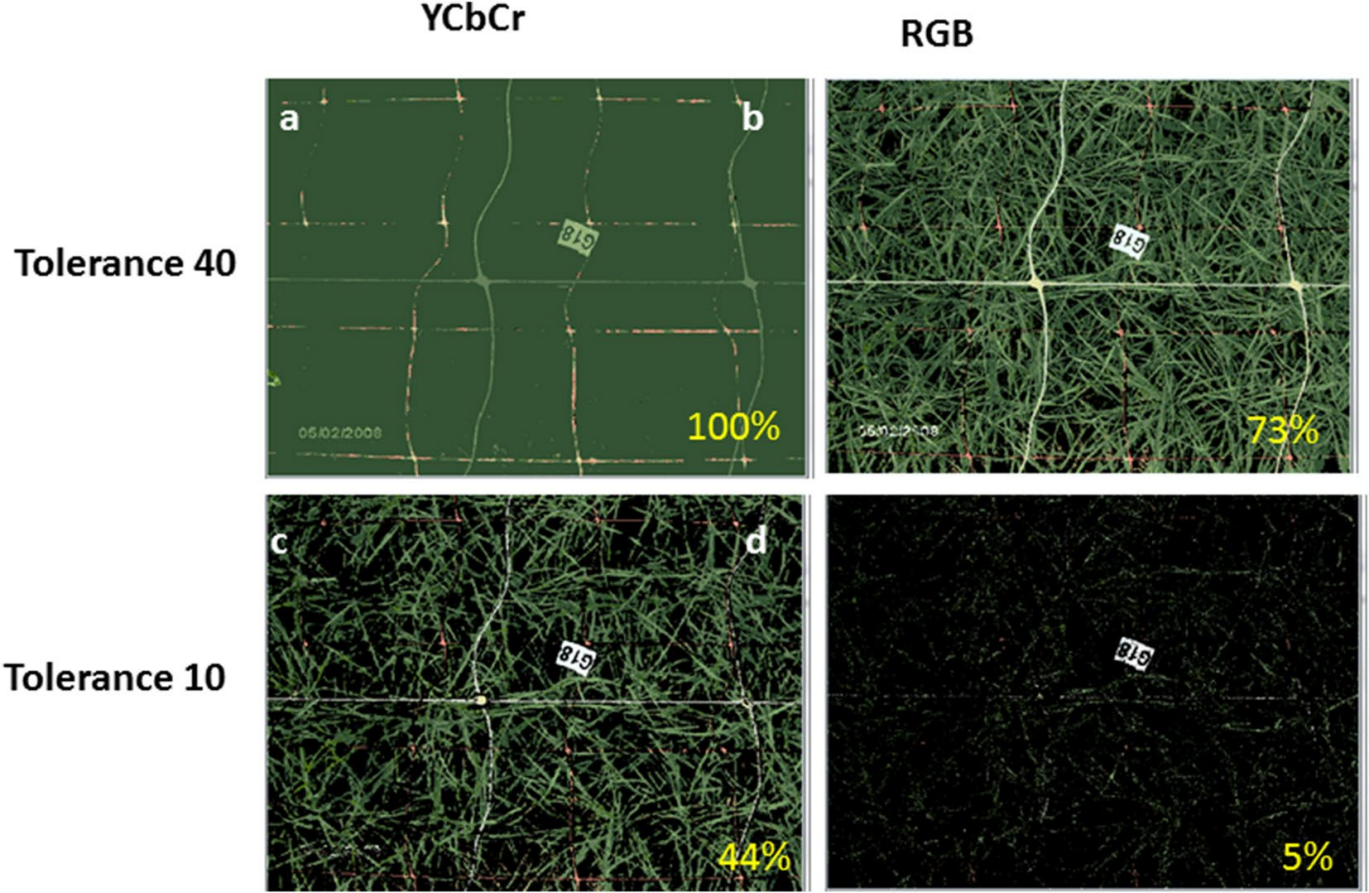
Tolerance and comparison metrics: tolerance of 40 (a, b) would be oo high and would overestimate the coverage area as compared to the level of 10 under both methods. However with the level of 10 under RGB there is an extreme underestimation of the coverage area (d) as compared to YCbCr metric at the same tolerance level

A tolerance level of 30 under the RGB metric gave the best match and was used to calculate the ‘percentages of canopy’ ground cover for the entire dataset (Fig. 9). Different tolerance levels under the different color comparison metrics (YCbCr and RGB) gave very different results as shown in Fig. 10 and a suitable metric and its tolerance need to be determined according to the data-set. Using the YCbCr metric can cover a color range with fewer shades as it deemphasizes the brightness of the pixel and concentrates on its hue. Thus, a single selection can cover a range of shades brightness having the same balance between R, G & B components.

### Case study 5—Tissue culture characterization

We now present an example of the routine use of *CoverageTool* in the characterization of tissue culture experiment in our lab.

**Fig. 11:**
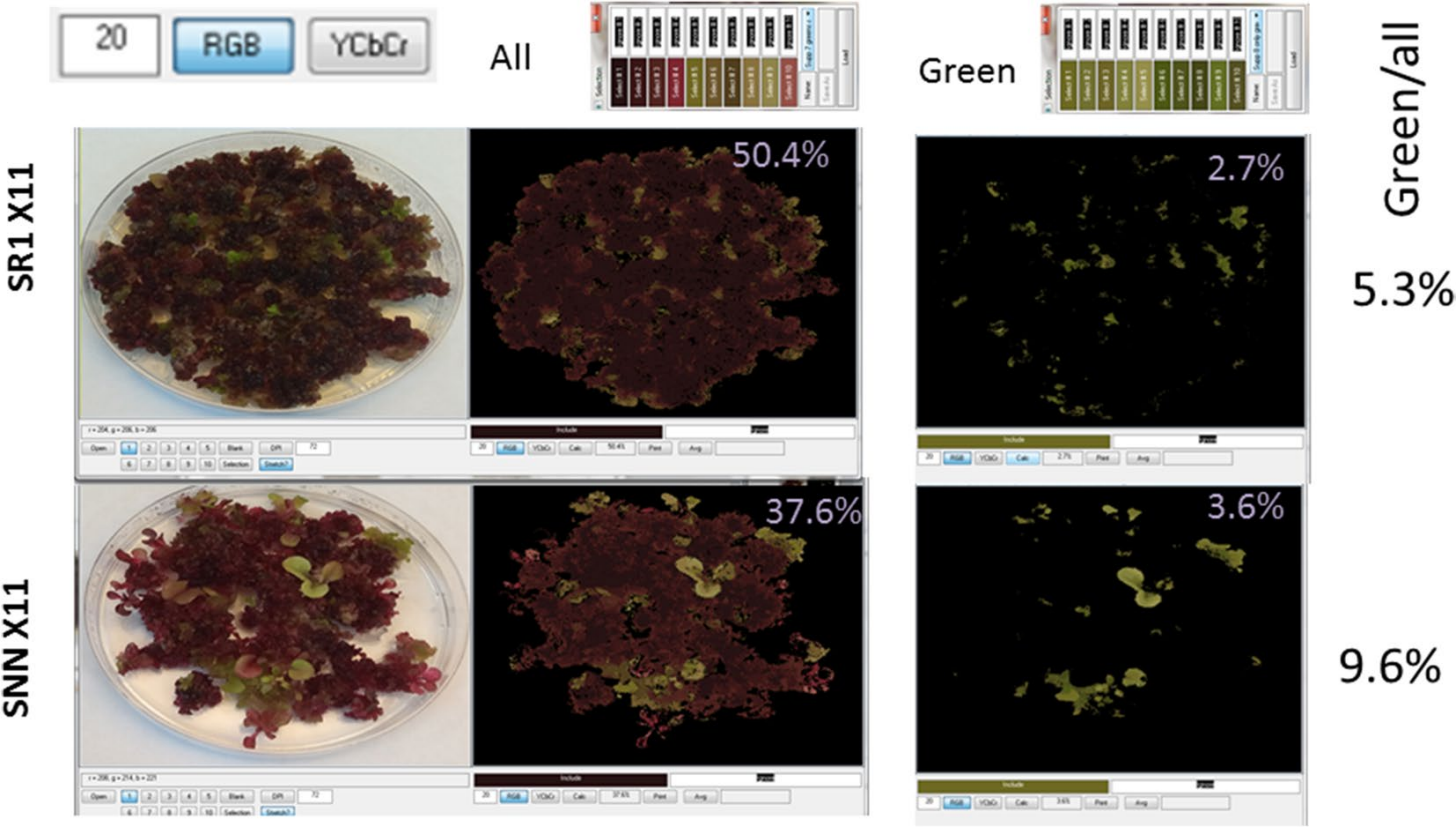
Tissue culture color analyses: *Nicotiana tabacum* SR1 and SNN regenerated calli transformed with the X11 construct vector. Shades of color representing plant tissue including reds and greens were selected to be taken into account as area coverage percentage. After calculating the total coverage in %, another color selection was made, which this time included only green shades. Then it was possible to calculate the ratio of green/total area for a given plate

For the characterization of the expression of the color marker in transgenic *Nicotiana tabacum* SR1 and SNN lines, we transformed sterile leaves with the pX11 vector that expresses a red-violet color in cells [13] using the A. *Tumefaciens* EHA105 strain. This vector overexpressing cytochrome P450 CYP76AD1, DOPA 4, 5-dioxygenase (BvDODA1) and cyclo-dopa 5-O-glucosyltransferase (cDOPA5GT), causes the development of red-violet calli or shoots, typically observed within 1–2 weeks of cultivation in tissue culture (Fig. 11). Transformation protocol and media are detailed in Additional file 9.

After several ‘generations’ cutting and isolating such red calli/shoots we observed there were “green escapes”, which were molecularly validated as silencing of X11 expressed genes. The specific mechanism is yet to be explored; however, *CoverageTool* was found as a valuable tool for the quantification the green areas in a Petri regeneration plates (Fig. 11 and Additional files 10, 11).

As a first step, the series of photos was cropped using MSPaint, then, saved as a 24-bit BMP files. The same pattern of cropping was applied, leaving the petri dish with a minimum background (Figs. 1, 11). Then, shades of color representing plant including reds and greens were selected to be taken into account as area coverage. Several images representing also the extremities of the set were sampled to ensure that all needed colors were sampled. Getting the best selection, which is subjective, may require several iterations (Additional file 4). However, even if the optimal selection is not achieved, the results will still be meaningful—providing that it was it applied uniformly across the data set. After calculating the total coverage in percent, we made another color selection, which this time included only the spectrum of green shades. Then it was possible to calculate the ratio of green/total area for a given plate, and compare between plates in our next experiments (Fig. 11).

## Discussion

A wide variety of tools and software were developed to address visual quantification of different biological specimens. However, these tools are frequently developed for a specific context, making them unsuitable for other applications [1]. More flexible software solutions, that provide a framework adaptable to multiple experimental designs recently developed, such as Rosette Tracker [14], rosettR [2], and “Do-It-Yourself” phenotyping system [15], which are specific for rosette plants (*Arabidopsis*) sometimes require special hardware. There are also numerous tools designated for root system analyses that rely on semi-automated root tracing [1]. In contrast, *CoverageTool* is a highly flexible tool that is not limited to an organ or a species and does not require any specific hardware, but a camera or scanner and a computer running Windows.

It is possible but rather complicated to filter according to a threshold using *ImageJ,* where sampling is limited to one ‘channel’ at a time. Using ImageJ, selection thresholding can be applied uniformly across an entire dataset; this, however necessitating a macro or loading the entire dataset as an image stack. *CoverageTool* provides better flexibility by enabling the sampling of 10 channels (to take into account as well as to ignore) simply by clicking different pixels to represent a custom dataset.. *ImageJ*, *PhotoShop* and other multi-MB software have very strong capabilities. The user has little idea how the ‘wand’ features work… For example, an area of the image is selected and its color distribution is somehow used to decide which pixels in the image are to be included in an object. Such a wand-selection needs to be applied manually and will be different in each image of the dataset—a process that lacks any uniformity, precluding statistically correct inter-image area ratio assessments. The tolerance for pixel inclusion and the decision algorithm are not clear. *CoverageTool* enables selecting a single pixel color for a selection channel and the algorithm of inclusion of colors similar to it is straightforward and can be applied uniformly to the entire dataset.

The results obtained using the *CoverageTool* and *ImageJ* (Table 1 and Fig. 3) validate Coverage’s results. However, obtaining these results using *CoverageTool* was significantly less time-consuming and the inter-image area ratios have better statistical significance than those of *ImageJ* due to the uniformity of application of thresholding. In addition, the selection capabilities of ImageJ do not provide the level of detail afforded by *CoverageTool*. *CoverageTool* allows 10 different shades, which would more likely fully segment objects as compared to *ImageJ*’s default thresholding. In addition, it allows more complex biological parameters to be quantified, such as senescence processes (as in case study 3) as well as simple efficient noise elimination (as in case study 4).

*CoverageTool* is suitable for the calculation of ground area coverage by a wheat canopy [12], for leaf area calculations [11], root surface area [9]—as well as for senescence rate calculations in barley [10] and any application that requires visual analyses (Additional file 12).

*CoverageTool* is a simple yet powerful tool for phenotyping, and was proven efficient in quantifying a variety of plant characteristics as summarized in Additional file 12: Table S1. Although developed and shown useful for the needs of the plant research community, it may also prove valuable for image analysis in various fields, other than plant phenotyping. The user needs to find the most suitable way to use *CoverageTool* for other applications by trial and error. Once mode of work and colors are selected, it takes about 35 min to process a set of 200 images (10 s/image). In summary, *CoverageTool* was found helpful in various fields of research, from tissue culture to the field work.

## Additional files

**Additional file 1.** *CoverageTool* ‘Coverage.exe’.

**Additional file 2.** Instructions for the use of *CoverageTool* ‘Coverage.exe’ (a Word doc).

**Additional file 3.** ‘Coverage.exe’ User Manual (a PowerPoint doc).

**Additional file 4.** A video documenting the analysis of leaf projected surface area using ‘Coverage.exe’ (an MP4 doc).

**Additional file 5.** Wheat scanned leaf blades, 24-bit BMP file.

**Additional file 6.** Wheat root scanned image, 24-bit BMP file.

**Additional file 7.** Barley shoot image, 24-bit BMP file.

**Additional file 8.** Wheat plot from above, early ground cover, 24-bit BMP file.

**Additional file 9.** Transformation protocol and culture media.

**Additional file 10.** *Nicotiana tabacum* SR1 transformer with X11 calli, cropped, 24-bit BMP file.

**Additional file 11.** *Nicotiana tabacum* SNN transformer with X11calli cropped, 24-bit BMP file.

**Additional file 12.** Summary of the different case-studies: image type, scale, Supp. file, phenotyping target, parameter calculated, units, sample to ‘coverage’ and/or to ‘ignore’, tolerance, RGB/YCbCr metric, and references.

## Data Availability

*CoverageTool* software and it’s additional files are in Additional files 1, 2, 3, 4, 5, 6, 7 and 8. Project name: *CoverageTool* Project home page: https://github.com/lianneovnat/CoverageTool.git Operating system(s): MS Windows: XP, Win7, Win10 etc. Programming language: “C” with WIN32 (Visual Studio 2008 Express Edition) Other requirements: Visual Studio 2008 Redistributal (or above) License: GNU.
