# Supplementary material for: CoverageTool: A semi-automated graphic software: applications for plant phenotyping
Source: Plant Methods. 2019 Aug 6;15:90. doi: 10.1186/s13007-019-0472-2 (PMC6683572; doi:10.1186/s13007-019-0472-2)
Supplement: Supplementary file 2 — Additional file 2. Instructions for the use of CoverageTool ‘Coverage.exe’ (a Word doc). [file 13007_2019_472_MOESM2_ESM.docx]

**Supp. 2**

***Instructions***

Instructions to use *CoverageTool* can be found also in supplementary file 3. After preparation of the dataset as described, run the program ‘Coverage.exe’. *CoverageTool* (Supp.1)*.*

**1**. Open your 24-bit BMP file. The image is initially displayed in the ‘source’ window on the left with 1 screen pixel per image pixel; if the image size exceeds the window pixel size, only part of the image is visible and the rest can be scrolled into view using the horizontal and vertical scrollbars. The image can be stretched/compressed to fit the window pixel size by clicking ‘Stretch’; however, when selecting pixel colors, it is recommended not to be in ‘stretched’ mode; in this mode a single screen pixel color is actually the average of several image pixels.

**2.** Choose between the RGB and YCbCr metrics.

**3**. Sampling the colors to include or ignore depends on the objects being assessed. In order to sample a channel (one of ten) to include, first make sure that the 'blank' radio button is not depressed, then, click on channel radio button, place your cursor on the pixel you would like to sample, and **left click**. Further channels may be sampled in the same manner. Next, it is possible to sample also channels to ignore, by clicking the channel radio button and then place your cruiser on the pixel you would like to sample to ignore (for each channel at a time) and **shift left click**.

**Note**: When calculating relative coverage in percent and the image includes patches or objects that are neither foreground nor background – these must be selected to ignore.

Not all 10 + 10 selections need be filled. The unused ones will have 'Include' and 'Ignore' with a black background. To reset a selection, first click the selection's button, then - right click anywhere in the image. Note: black is automatically ignored - so it need not be selected.

**4.** Set a tolerance level around 20 to start with, and later adjust according to the results.

**5.** Calculate by pressing the 'Calc' radio button. Having the 'Blank' button depressed - the area will be calculated in cm^2^; otherwise, the coverage percentage will be calculated.

**6.** Go to step 3, re-calculate and improve your selection as many times as needed.

**7.** Analyze several images to represent the dataset extremities, make sure your samples cover the entire dataset.

**8.** Upon finalizing the dataset’s customized selection, save it by clicking 'Selection'. This opens the selections dialog box, where the custom selection can be assigned a name and saved by clicking the ‘Save As’ button. To re-apply this selection again, open the selection box and re-load your saved settings.

**9.** Press the 'Print’ button to append a record to 'Coverage.txt' having the format:

<filename>, result, tolerance, <'RGB' or 'YCbCr'>, RGB_Include[1], RGB_Ignore[1], RGB_Include[2], RGB_Ignore[2], ... RGB_Include[10], RGB_Ignore[10].

**10.** The ‘Avg’ button - brings up a message box that displays the average RGB components for the foreground image pixels characterized by each of non-zero selections 1-10 and the weighted total averages for the RGB components of all the non-zero selections - and appends the records to 'avg.txt' (Supp. 2).

**11.** To erase background artifacts, depress 'blank', select an eraser brush width using the 10 radio buttons brush them out.

**12.** To exclude everything but the interior of a quadrilateral, position the cursor over its 4 vertices in turn clicking F1, F2, F3 & F4 in clockwise order; at top left click F1, at top right click F2, at bottom right click F3, at bottom left click F4.

**Note**: whenever ‘Calc’ or ‘Selection’ are clicked, the current selection is saved as default and will be loaded upon restart of the application.
