# Supplementary material for: CoverageTool: A semi-automated graphic software: applications for plant phenotyping
Source: Plant Methods. 2019 Aug 6;15:90. doi: 10.1186/s13007-019-0472-2 (PMC6683572; doi:10.1186/s13007-019-0472-2)
Supplement: Supplementary file 3 — Additional file 3. ‘Coverage.exe’ User Manual (a PowerPoint doc). [file 13007_2019_472_MOESM3_ESM.pptx]

## Slide 1
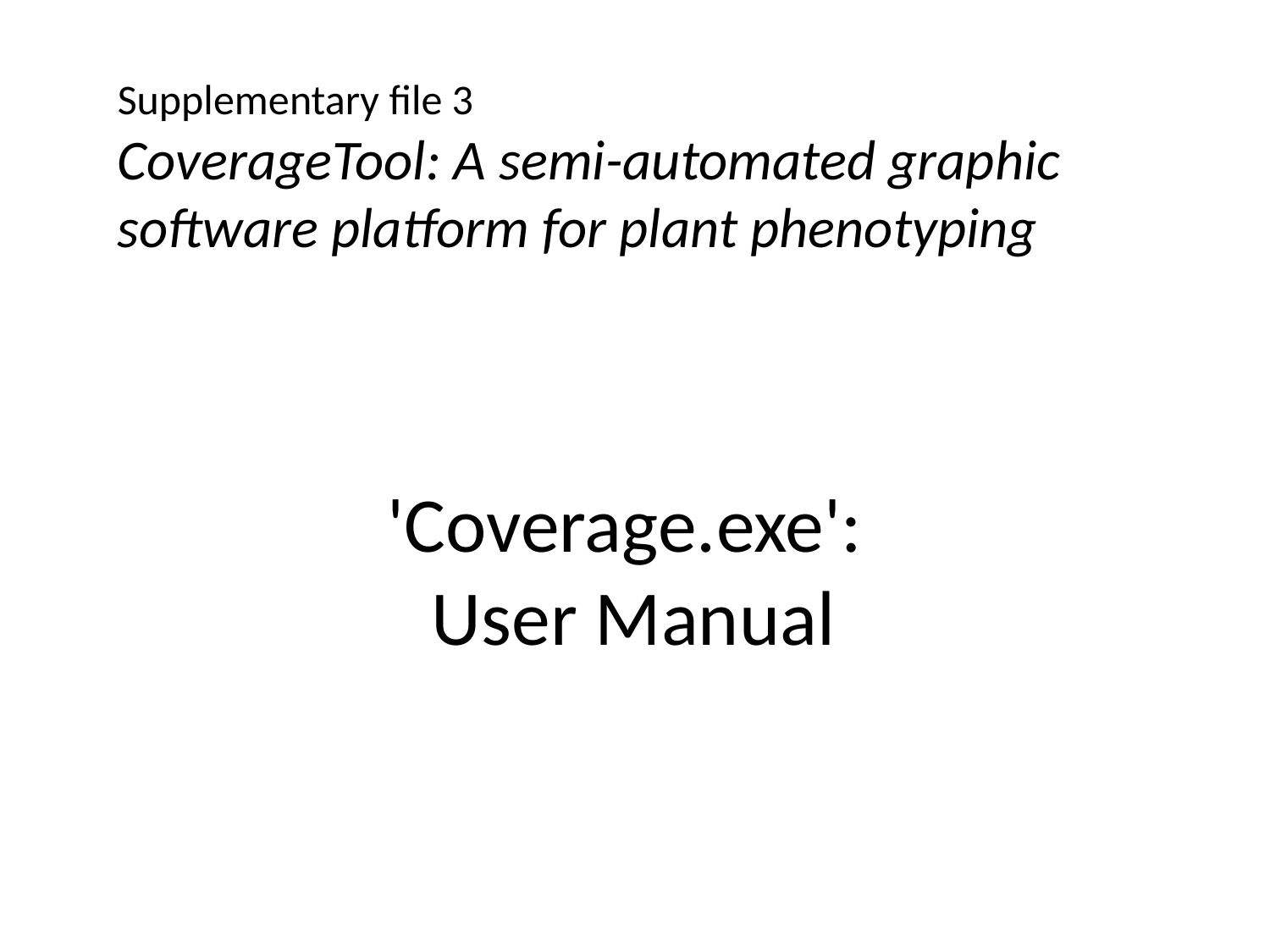

Supplementary file 3
CoverageTool: A semi-automated graphic software platform for plant phenotyping
# 'Coverage.exe': User Manual

## Slide 2
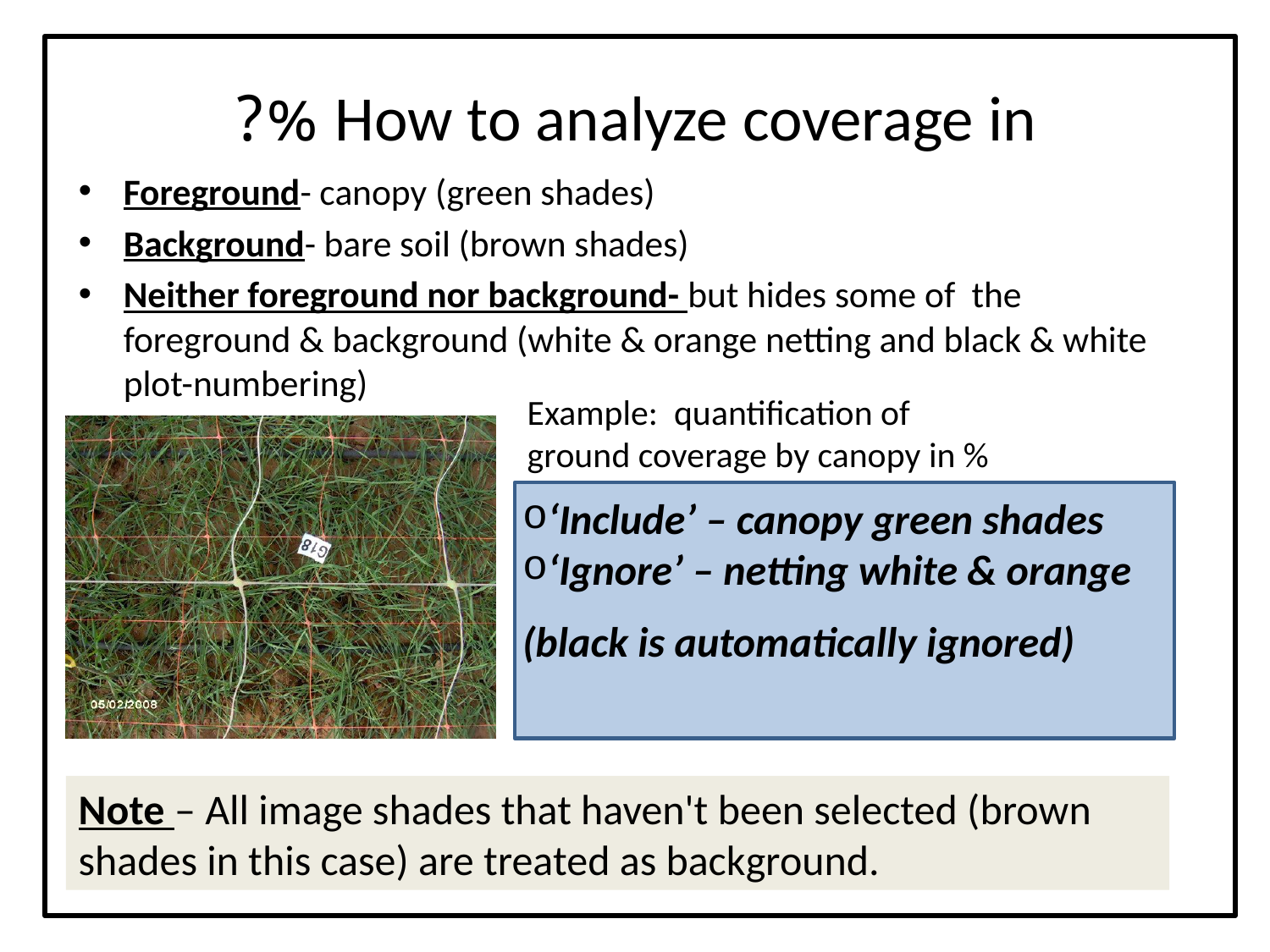

# How to analyze coverage in %?
Foreground- canopy (green shades)
Background- bare soil (brown shades)
Neither foreground nor background- but hides some of the foreground & background (white & orange netting and black & white plot-numbering)
Example: quantification of
ground coverage by canopy in %
‘Include’ – canopy green shades
‘Ignore’ – netting white & orange
(black is automatically ignored)
Note – All image shades that haven't been selected (brown shades in this case) are treated as background.

## Slide 3
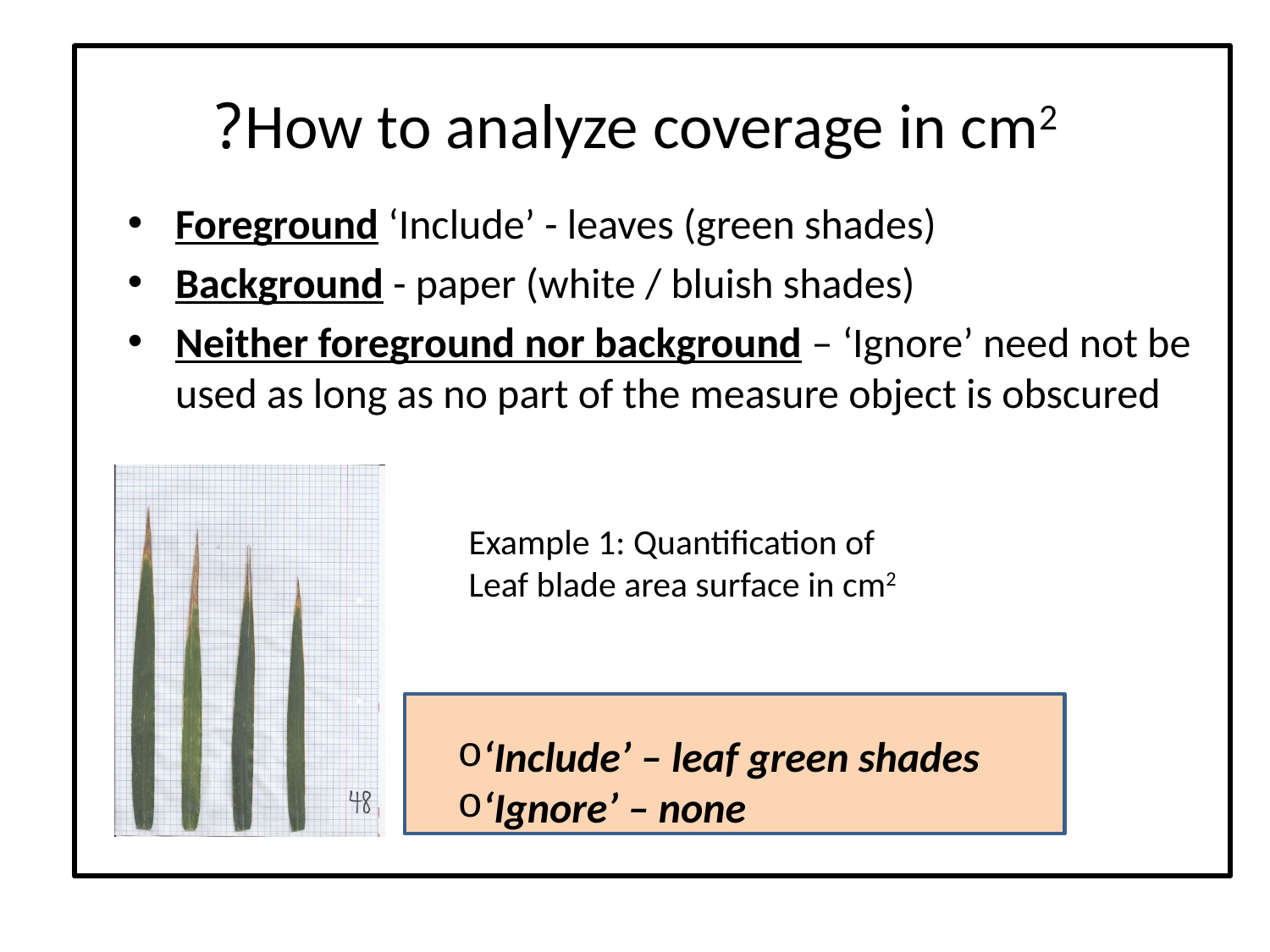

# How to analyze coverage in cm2?
Foreground ‘Include’ - leaves (green shades)
Background - paper (white / bluish shades)
Neither foreground nor background – ‘Ignore’ need not be used as long as no part of the measure object is obscured
Example 1: Quantification of
Leaf blade area surface in cm2
‘Include’ – leaf green shades
‘Ignore’ – none

## Slide 4
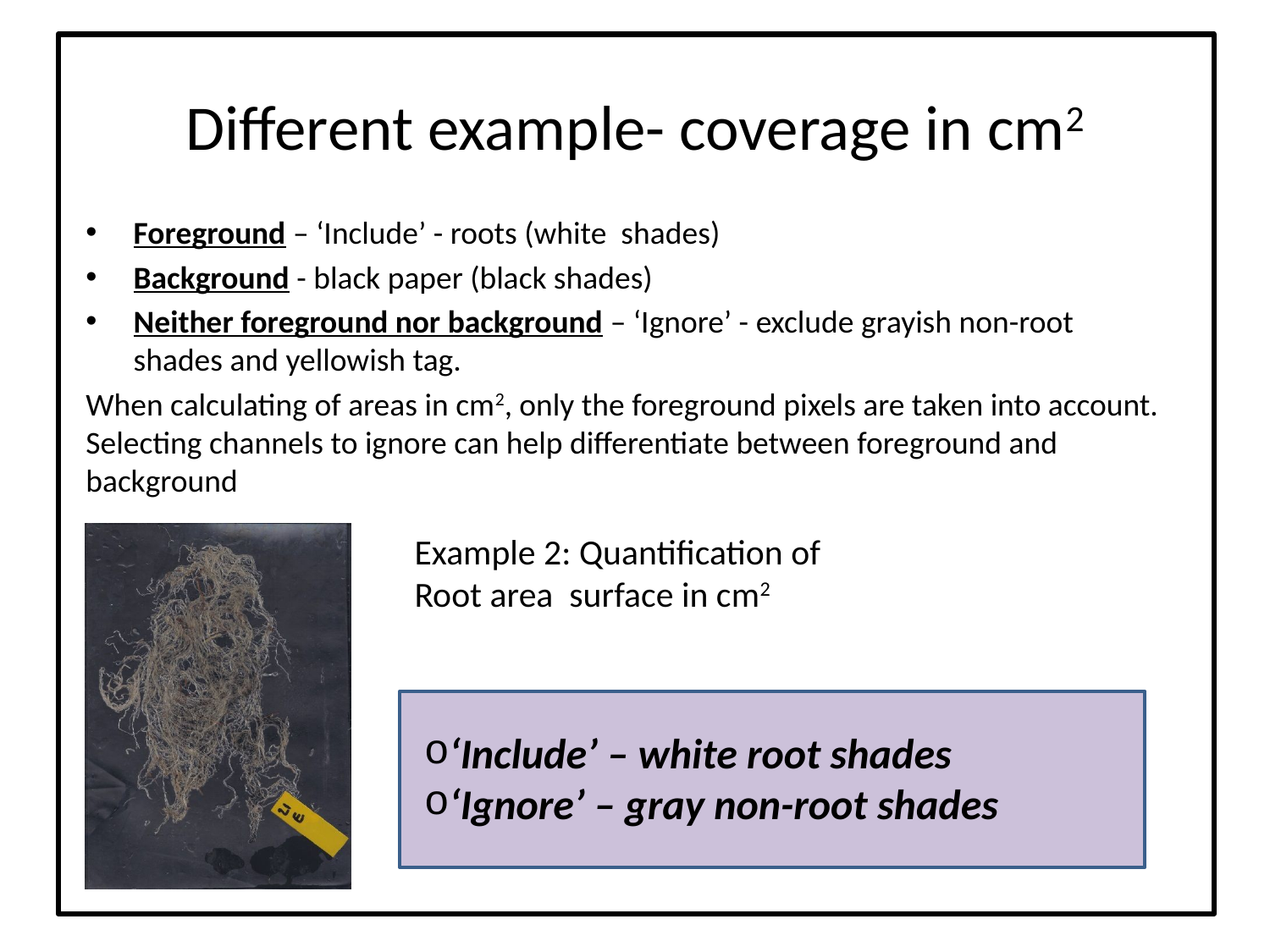

# Different example- coverage in cm2
Foreground – ‘Include’ - roots (white shades)
Background - black paper (black shades)
Neither foreground nor background – ‘Ignore’ - exclude grayish non-root shades and yellowish tag.
When calculating of areas in cm2, only the foreground pixels are taken into account. Selecting channels to ignore can help differentiate between foreground and background
Example 2: Quantification of
Root area surface in cm2
‘Include’ – white root shades
‘Ignore’ – gray non-root shades

## Slide 5
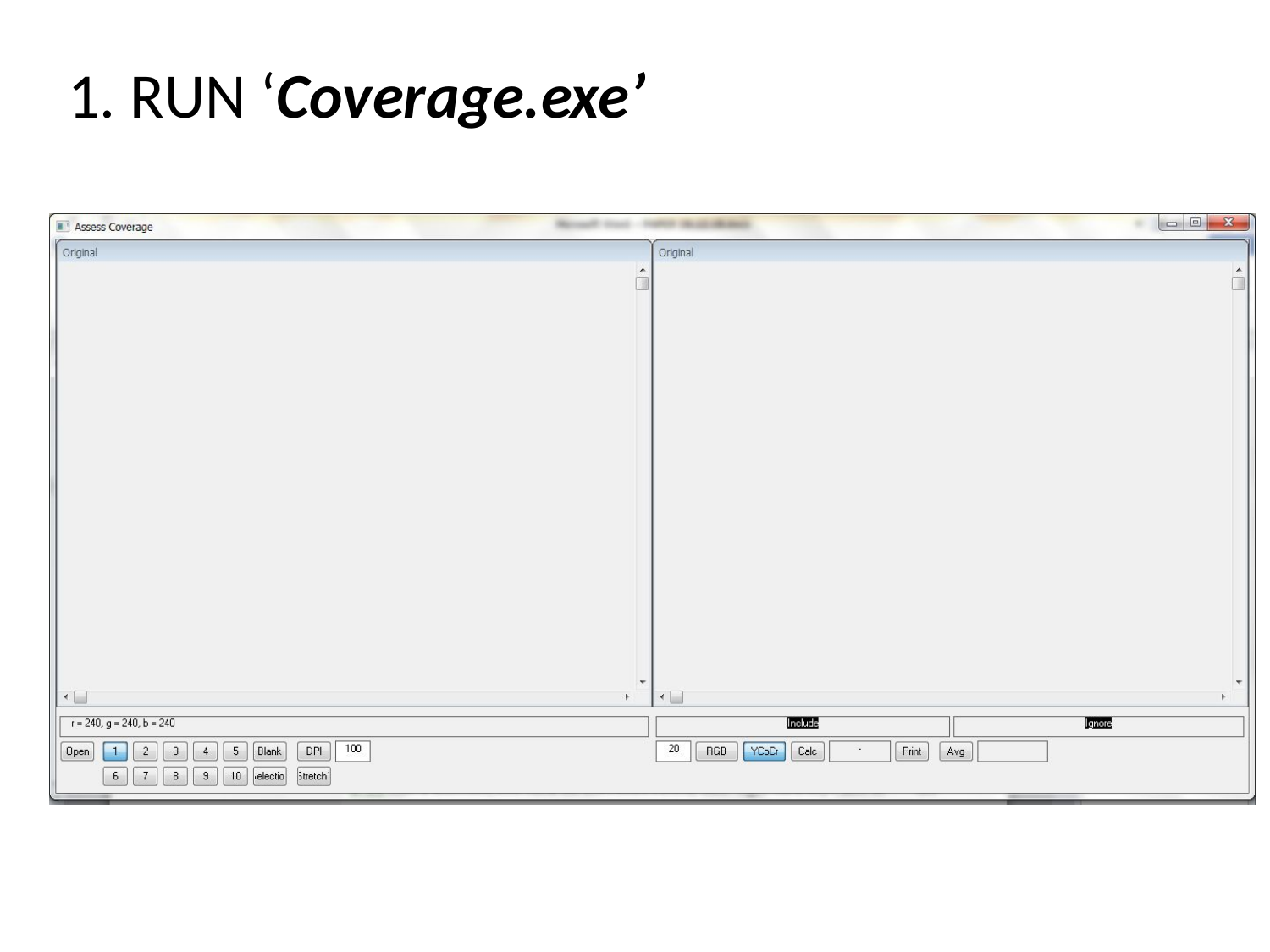

# 1. RUN ‘Coverage.exe’

## Slide 6
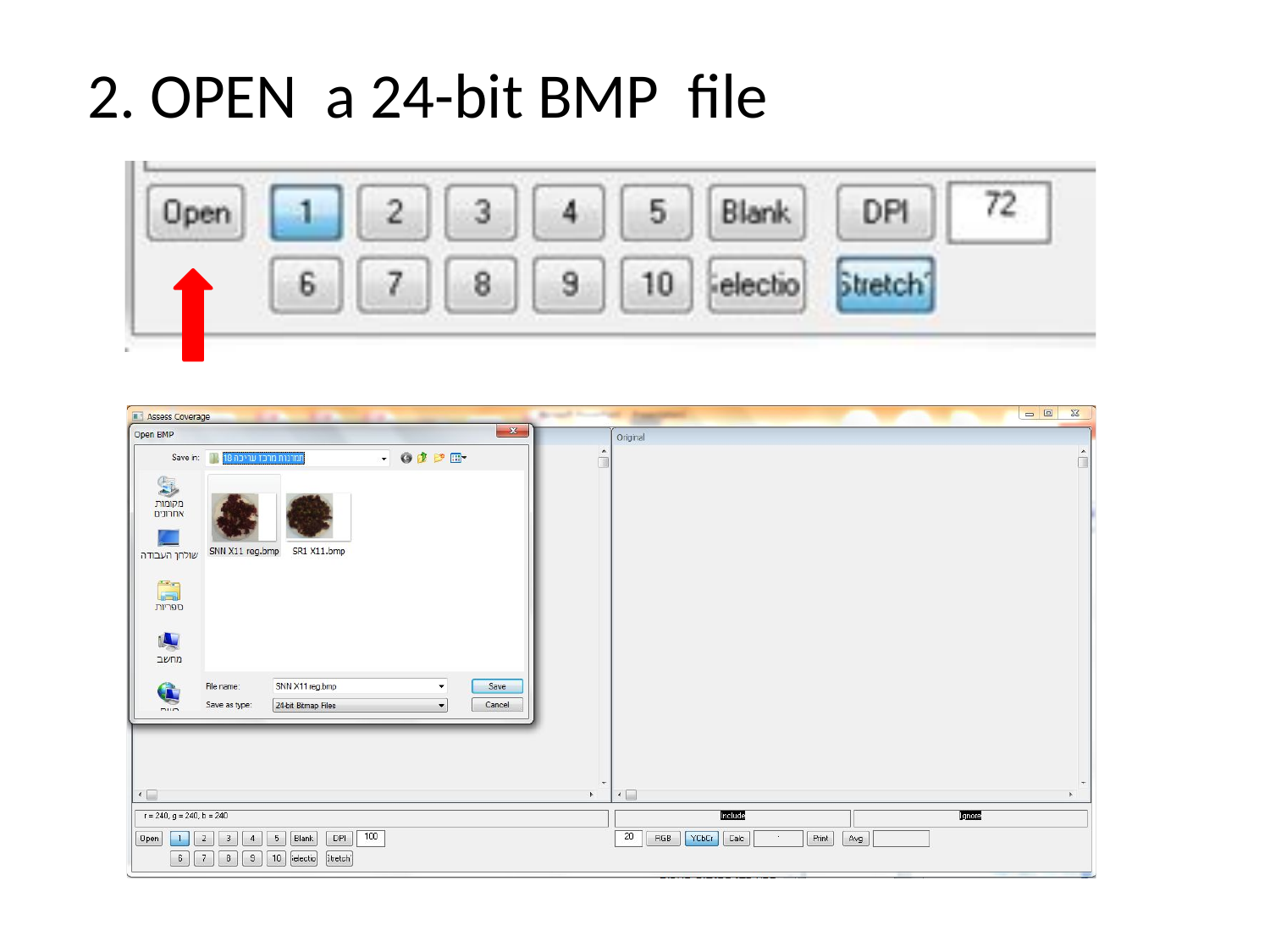

# 2. OPEN a 24-bit BMP file

## Slide 7
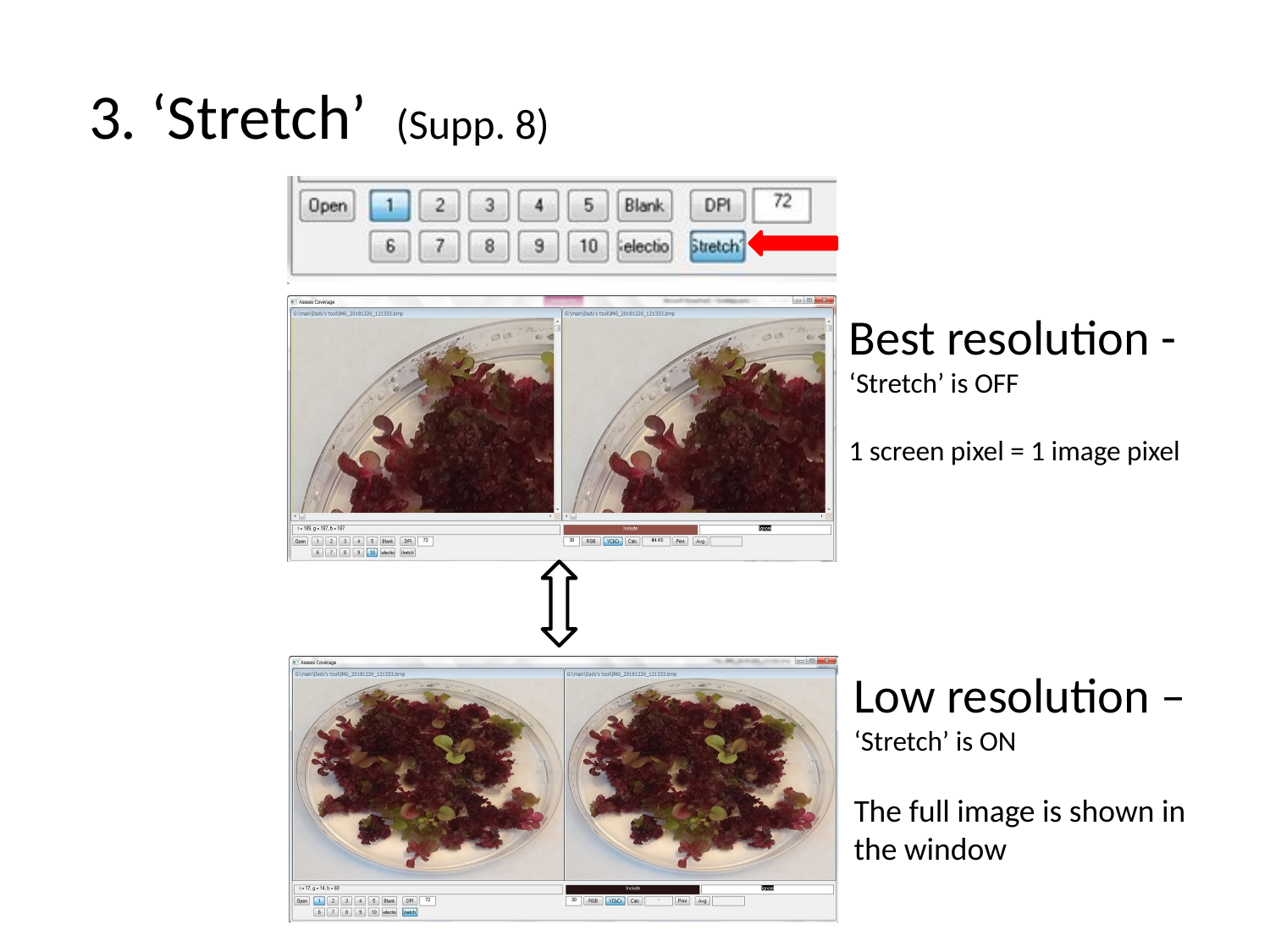

# 3. ‘Stretch’ (Supp. 8)
Best resolution -
‘Stretch’ is OFF
1 screen pixel = 1 image pixel
Low resolution –
‘Stretch’ is ON
The full image is shown in the window

## Slide 8
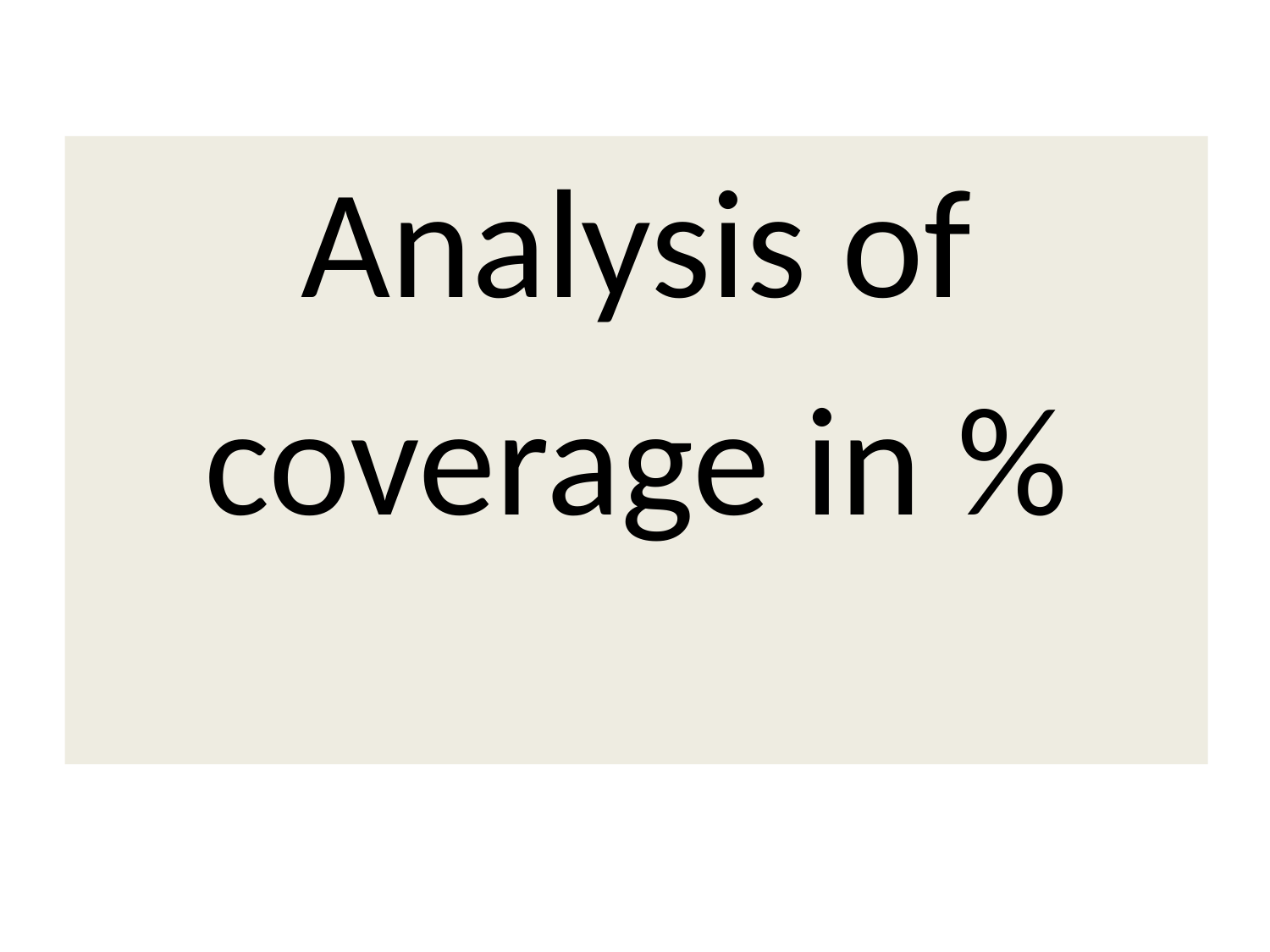

Analysis of
coverage in %

## Slide 9
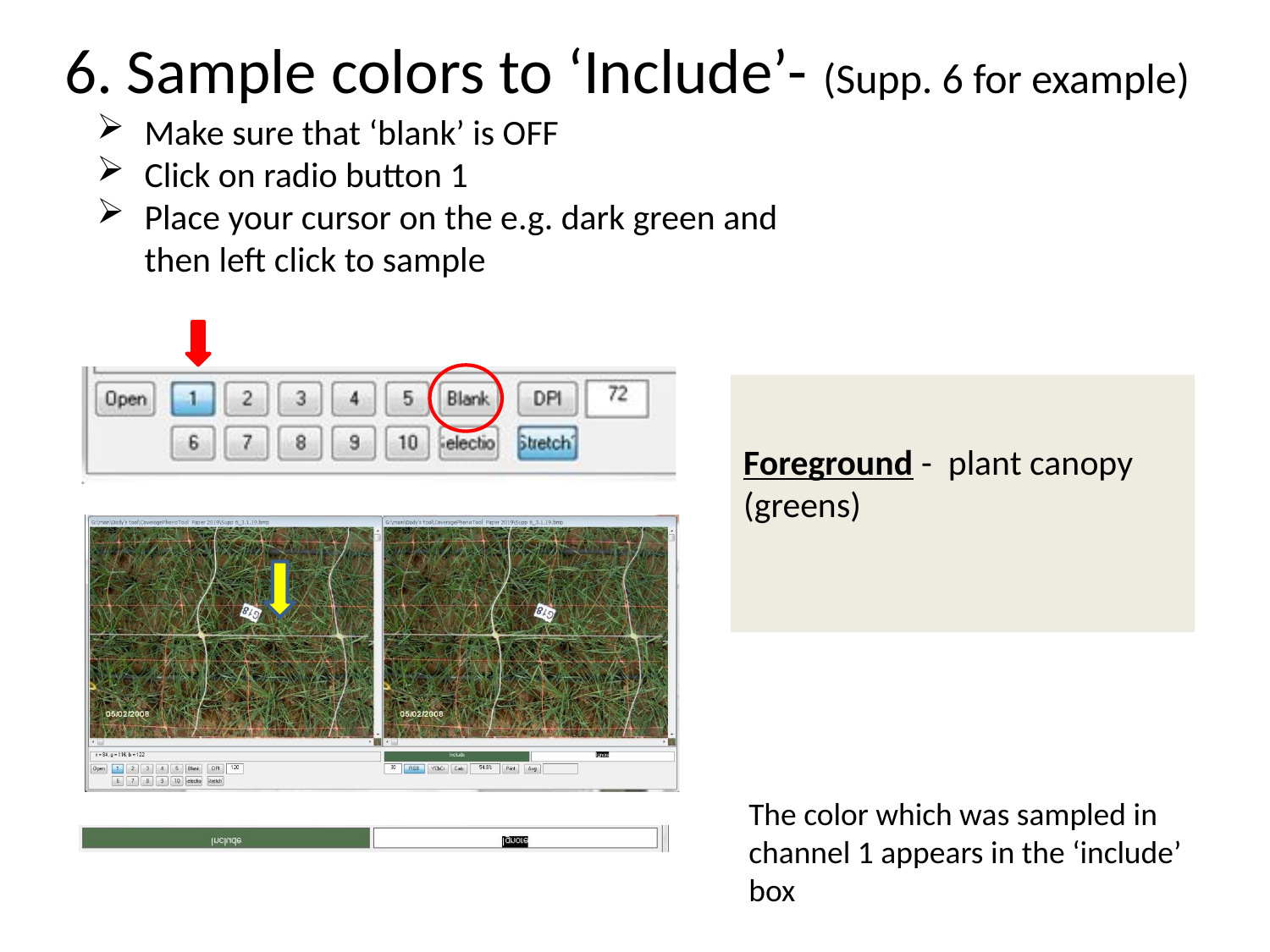

6. Sample colors to ‘Include’- (Supp. 6 for example)
Make sure that ‘blank’ is OFF
Click on radio button 1
Place your cursor on the e.g. dark green and then left click to sample
# Foreground - plant canopy (greens)
The color which was sampled in channel 1 appears in the ‘include’ box

## Slide 10
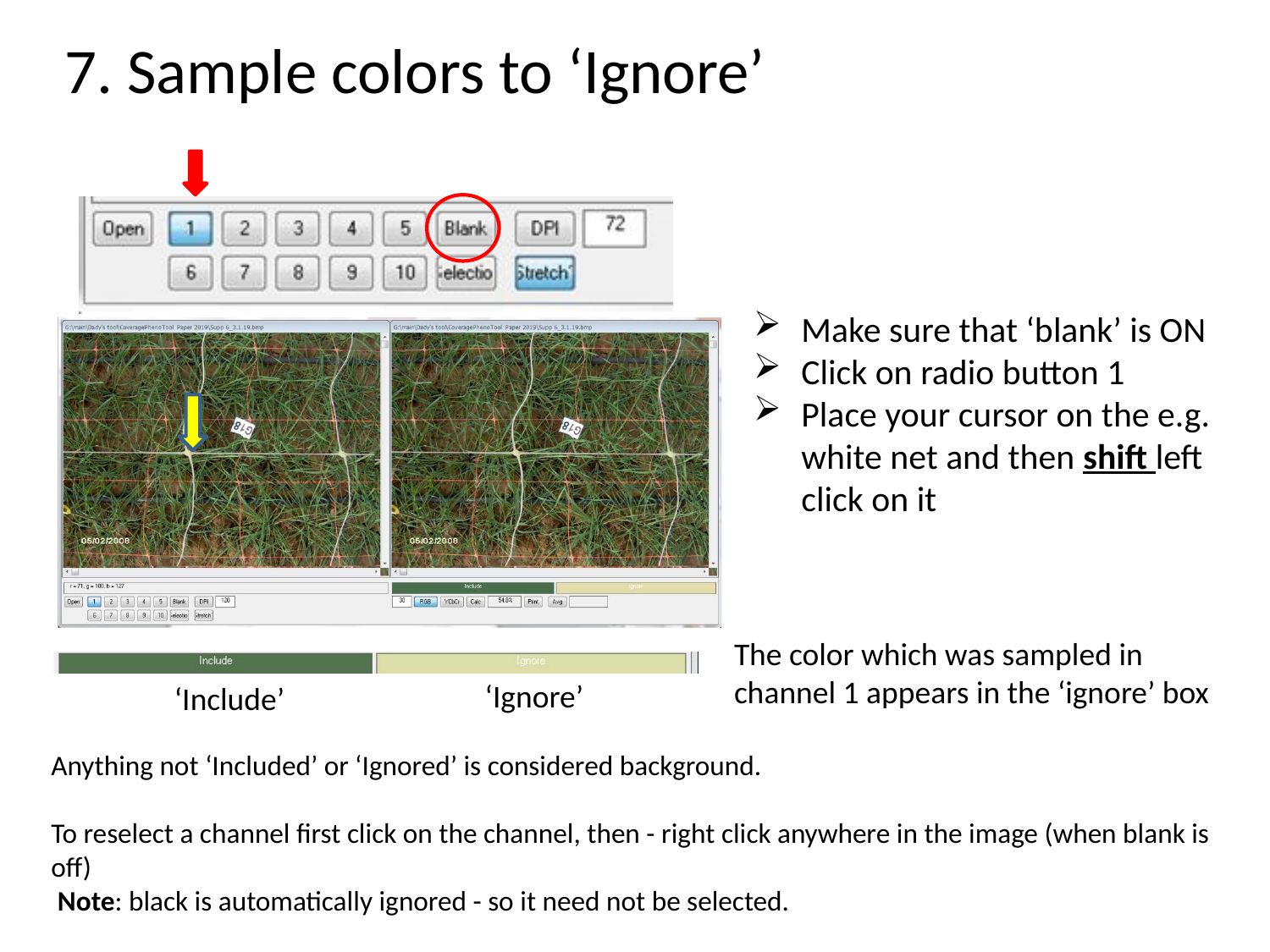

# 7. Sample colors to ‘Ignore’
Make sure that ‘blank’ is ON
Click on radio button 1
Place your cursor on the e.g. white net and then shift left click on it
The color which was sampled in channel 1 appears in the ‘ignore’ box
‘Ignore’
‘Include’
Anything not ‘Included’ or ‘Ignored’ is considered background.
To reselect a channel first click on the channel, then - right click anywhere in the image (when blank is off)
 Note: black is automatically ignored - so it need not be selected.

## Slide 11
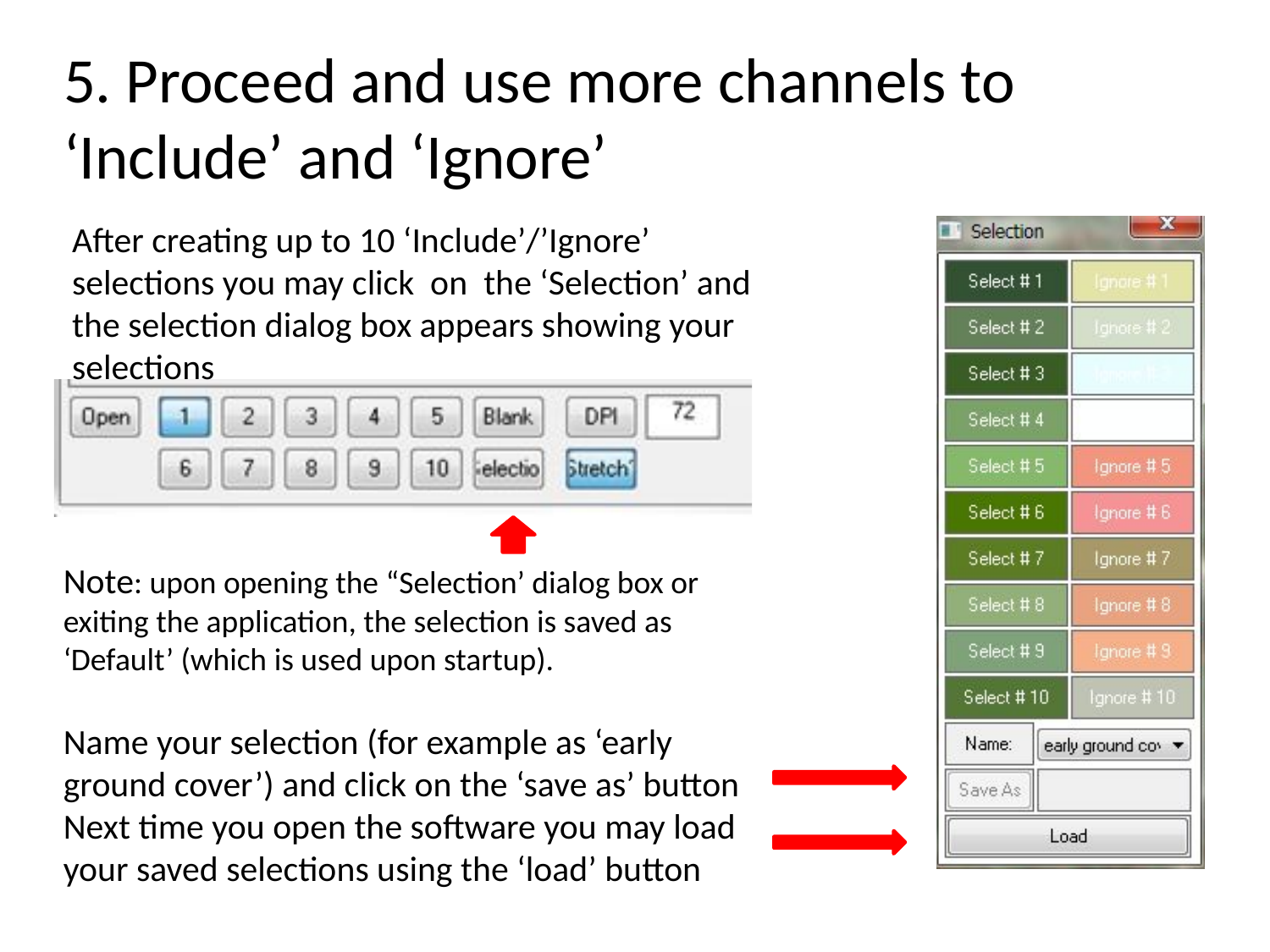

# 5. Proceed and use more channels to ‘Include’ and ‘Ignore’
After creating up to 10 ‘Include’/’Ignore’ selections you may click on the ‘Selection’ and the selection dialog box appears showing your selections
Note: upon opening the “Selection’ dialog box or exiting the application, the selection is saved as ‘Default’ (which is used upon startup).
Name your selection (for example as ‘early ground cover’) and click on the ‘save as’ button
Next time you open the software you may load your saved selections using the ‘load’ button

## Slide 12
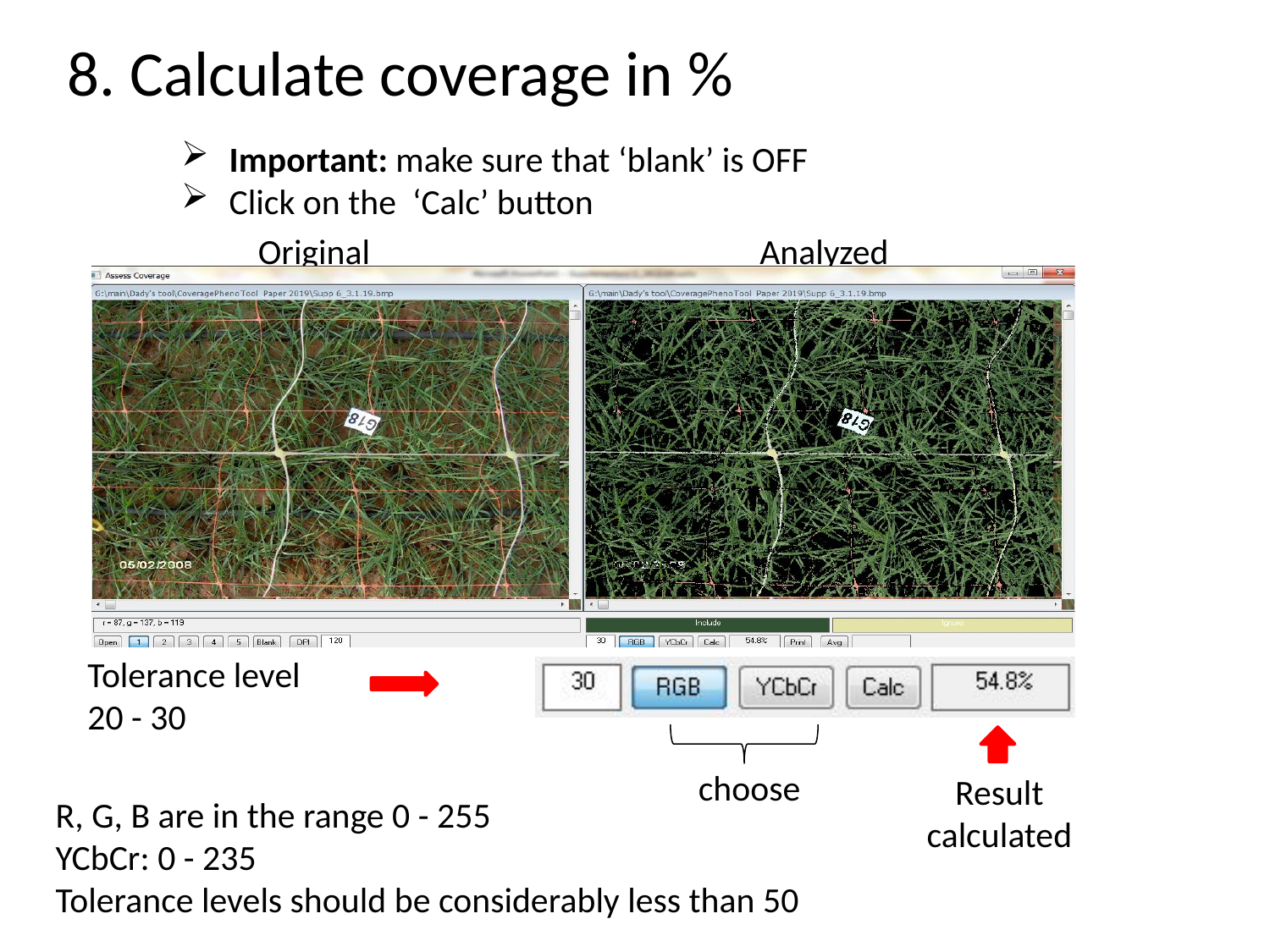

# 8. Calculate coverage in %
Important: make sure that ‘blank’ is OFF
Click on the ‘Calc’ button
Original
Analyzed
Tolerance level
20 - 30
choose
Result calculated
R, G, B are in the range 0 - 255
YCbCr: 0 - 235
Tolerance levels should be considerably less than 50

## Slide 13
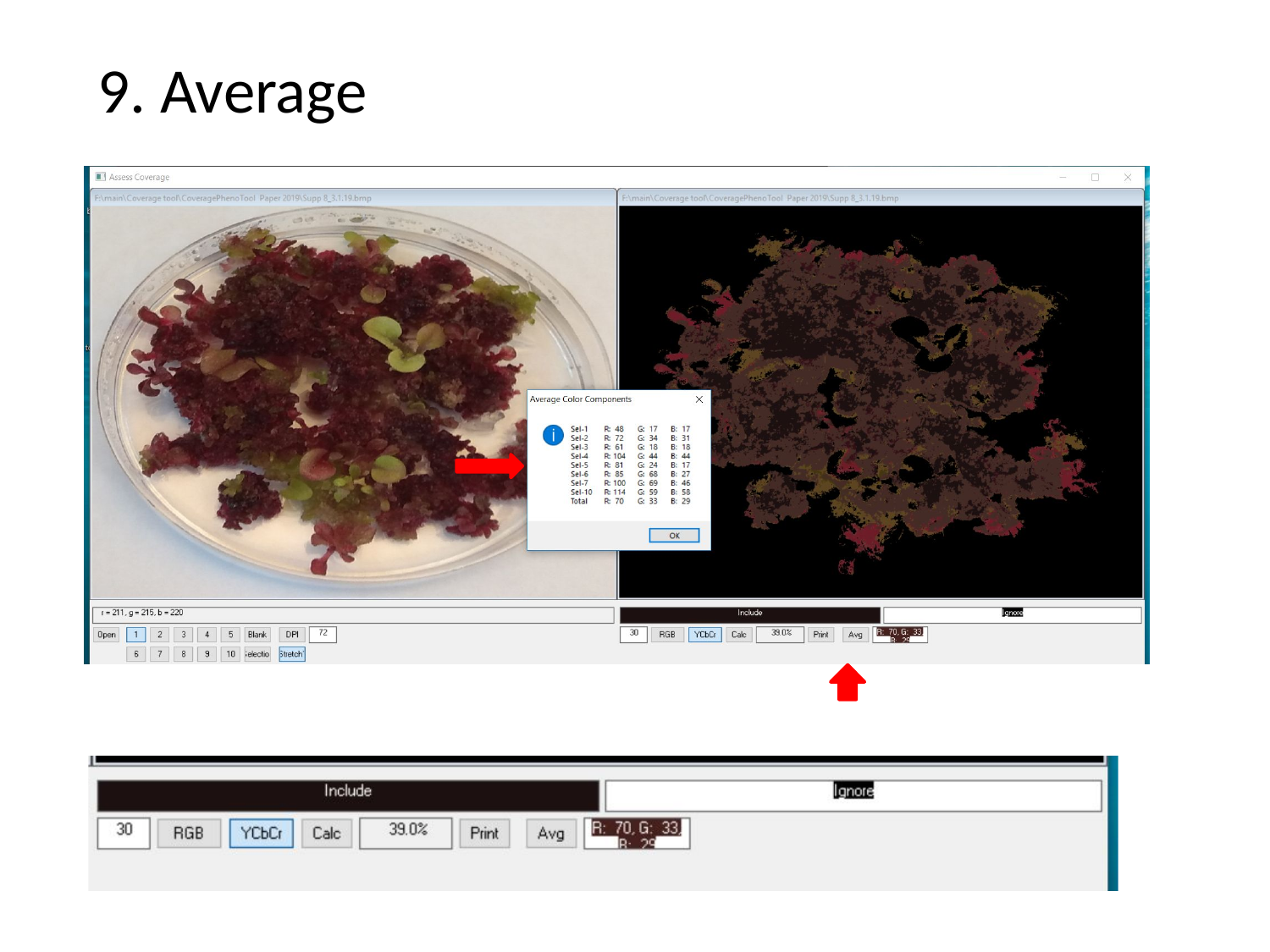

9. Average

## Slide 14
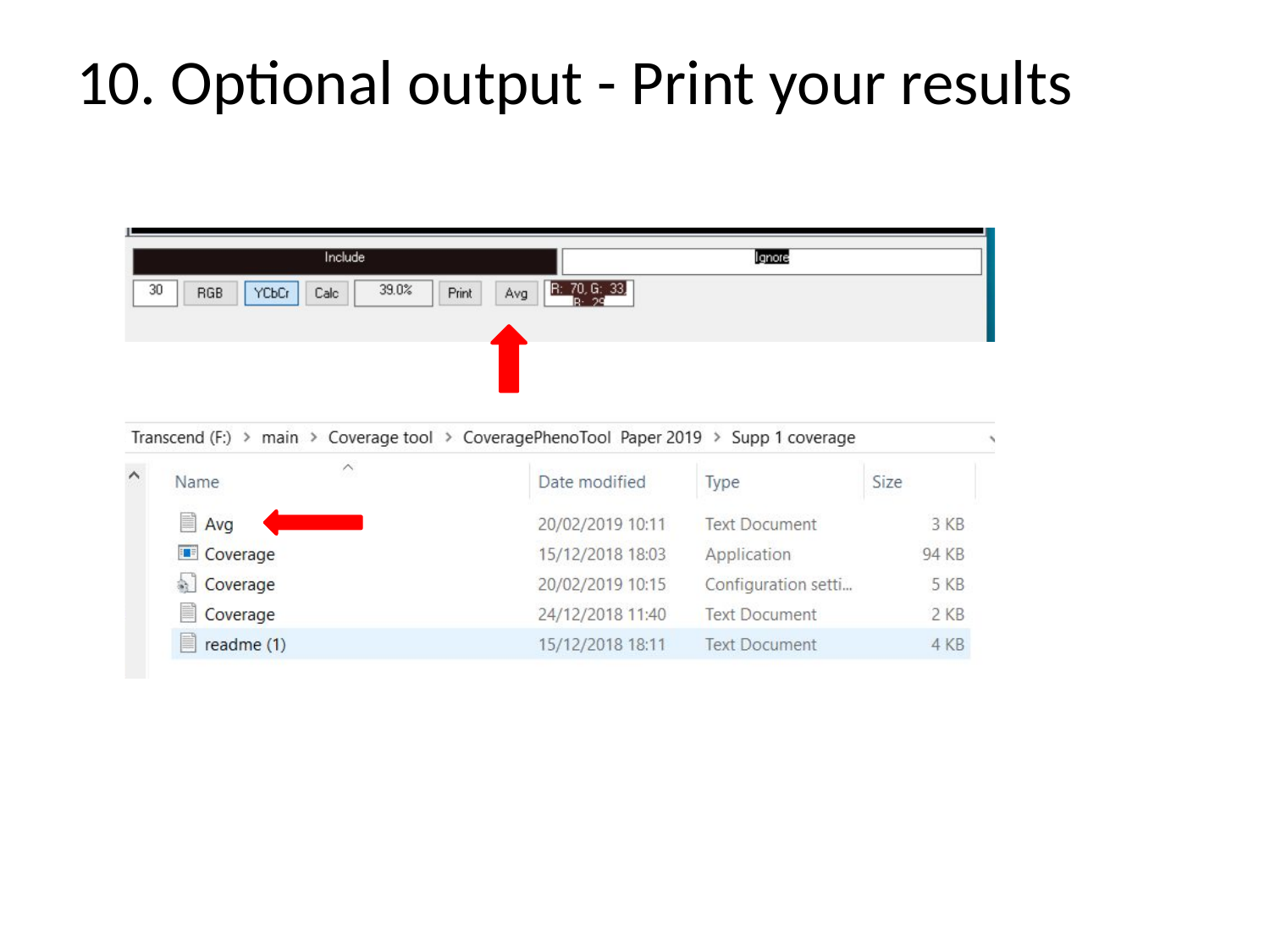

# 10. Optional output - Print your results

## Slide 15
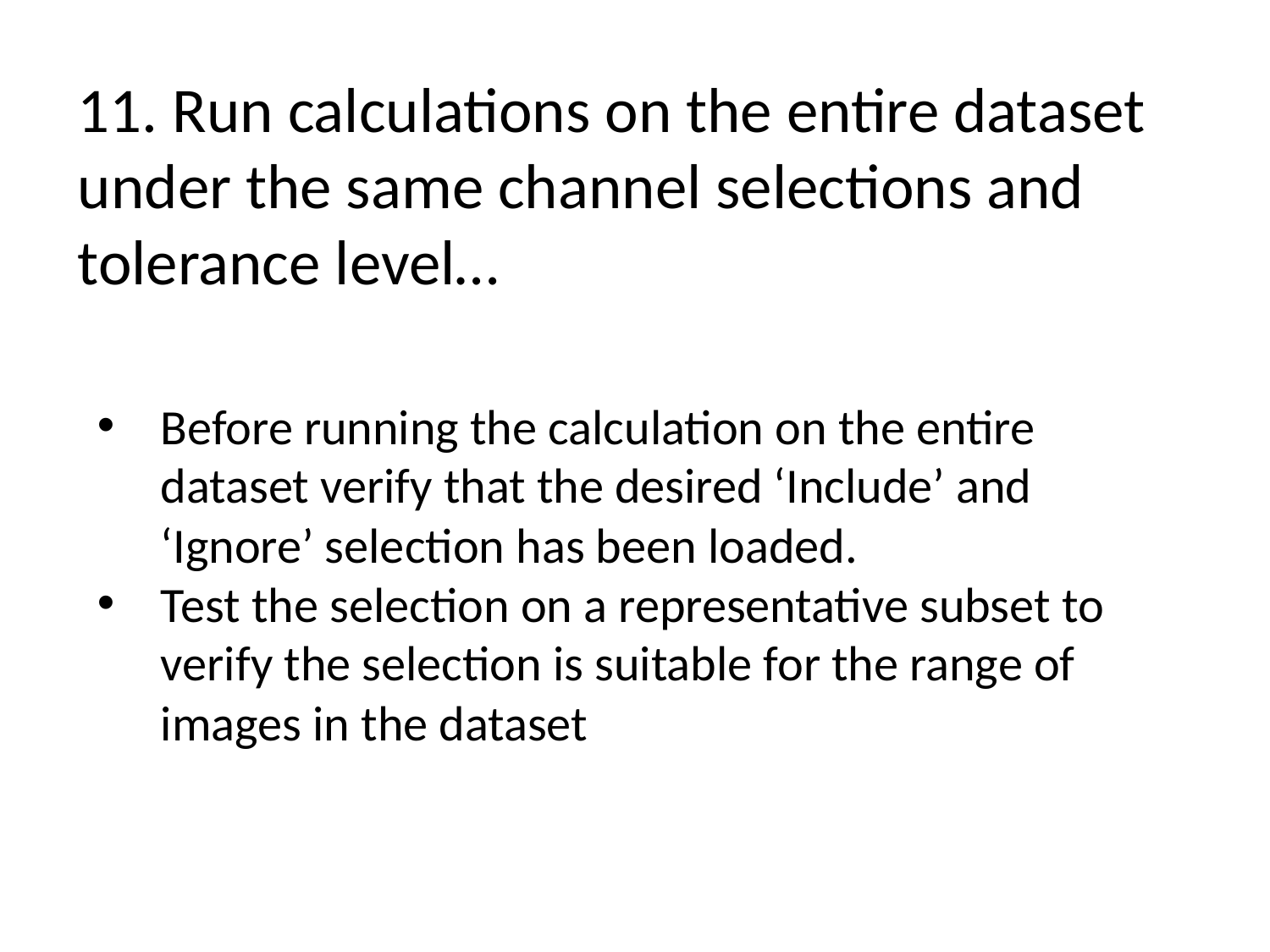

11. Run calculations on the entire dataset
under the same channel selections and tolerance level…
Before running the calculation on the entire dataset verify that the desired ‘Include’ and ‘Ignore’ selection has been loaded.
Test the selection on a representative subset to verify the selection is suitable for the range of images in the dataset

## Slide 16
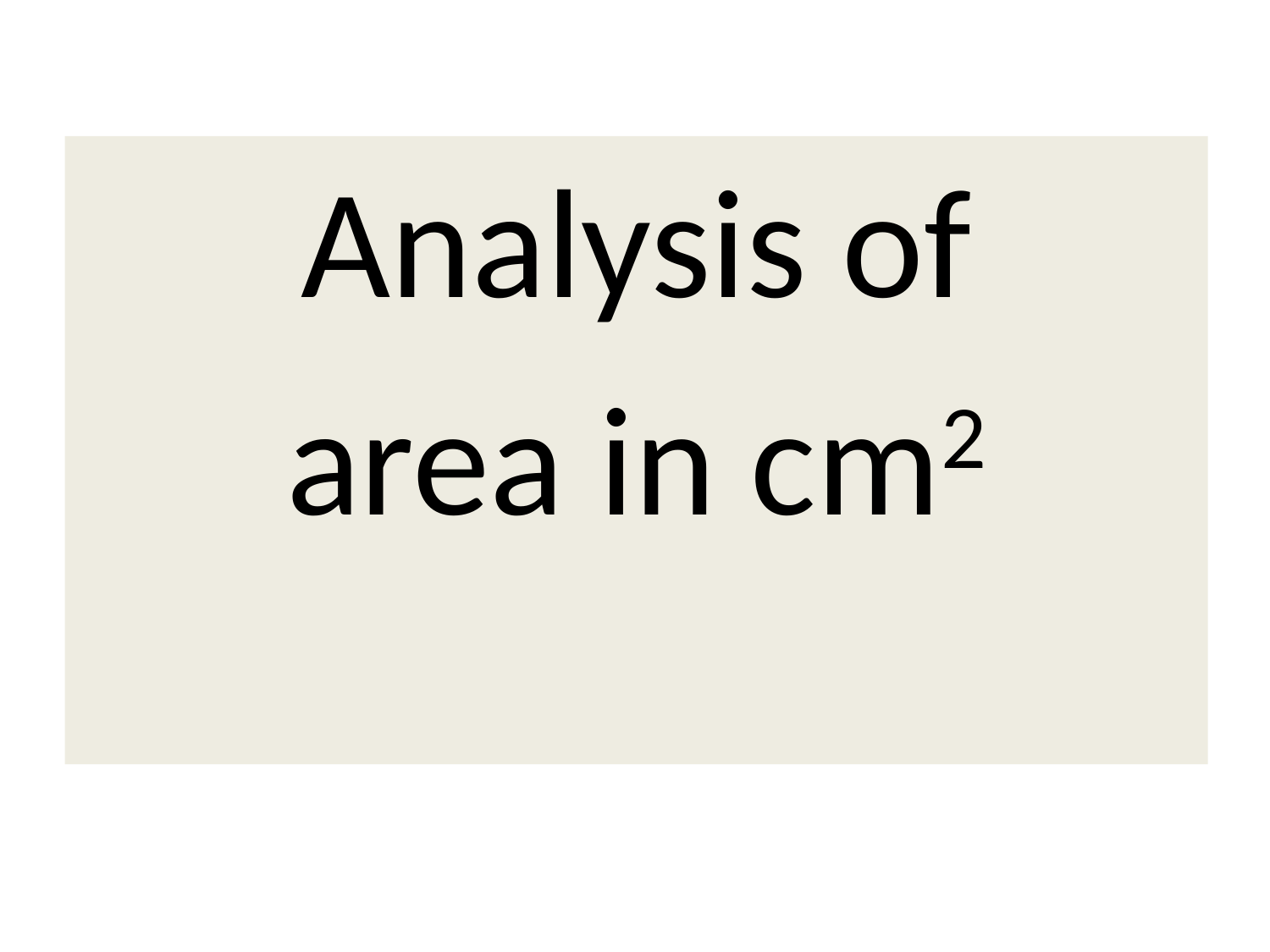

Analysis of
area in cm2

## Slide 17
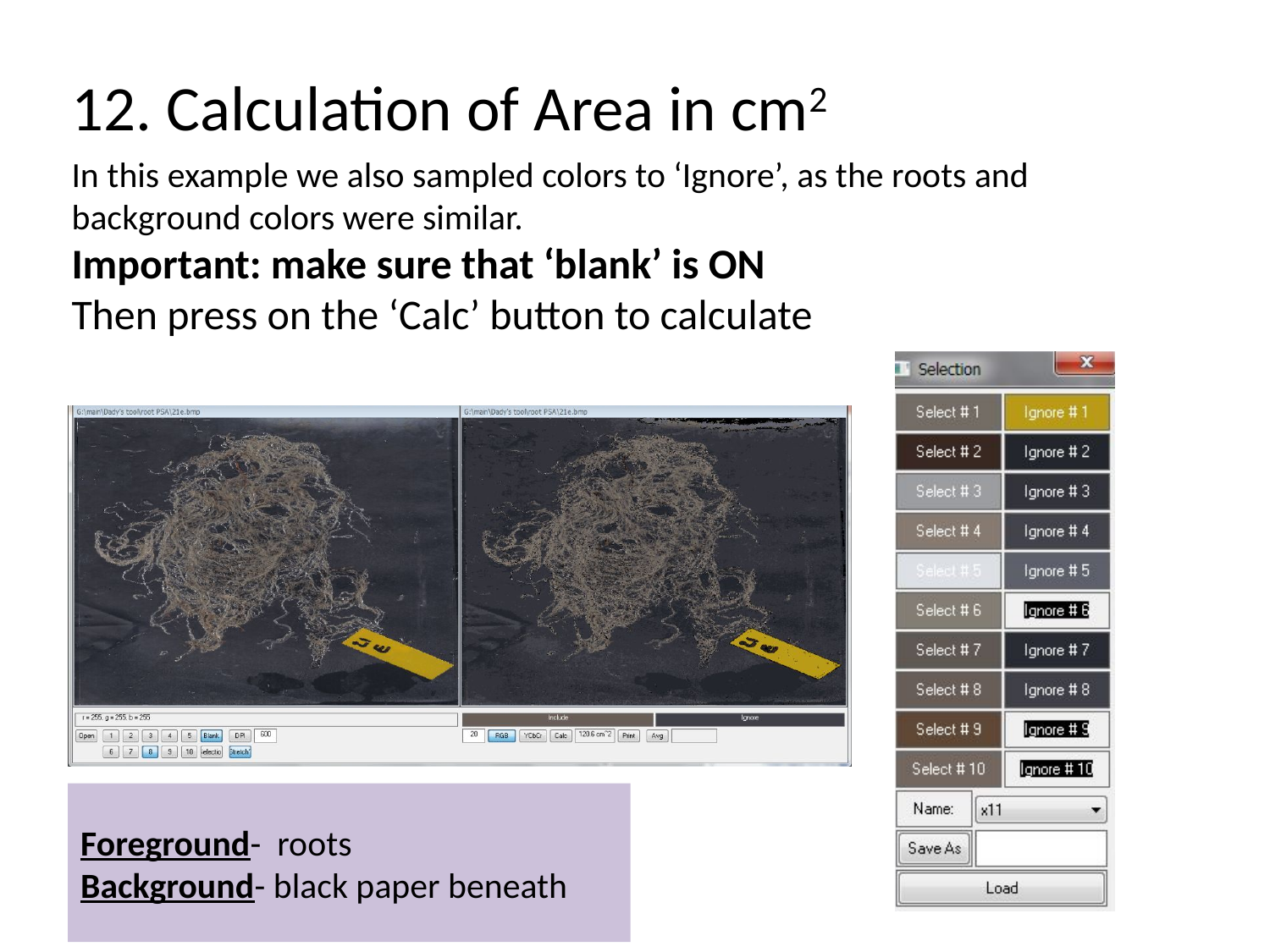

12. Calculation of Area in cm2
In this example we also sampled colors to ‘Ignore’, as the roots and background colors were similar.
Important: make sure that ‘blank’ is ON
Then press on the ‘Calc’ button to calculate
# Foreground- roots Background- black paper beneath

## Slide 18
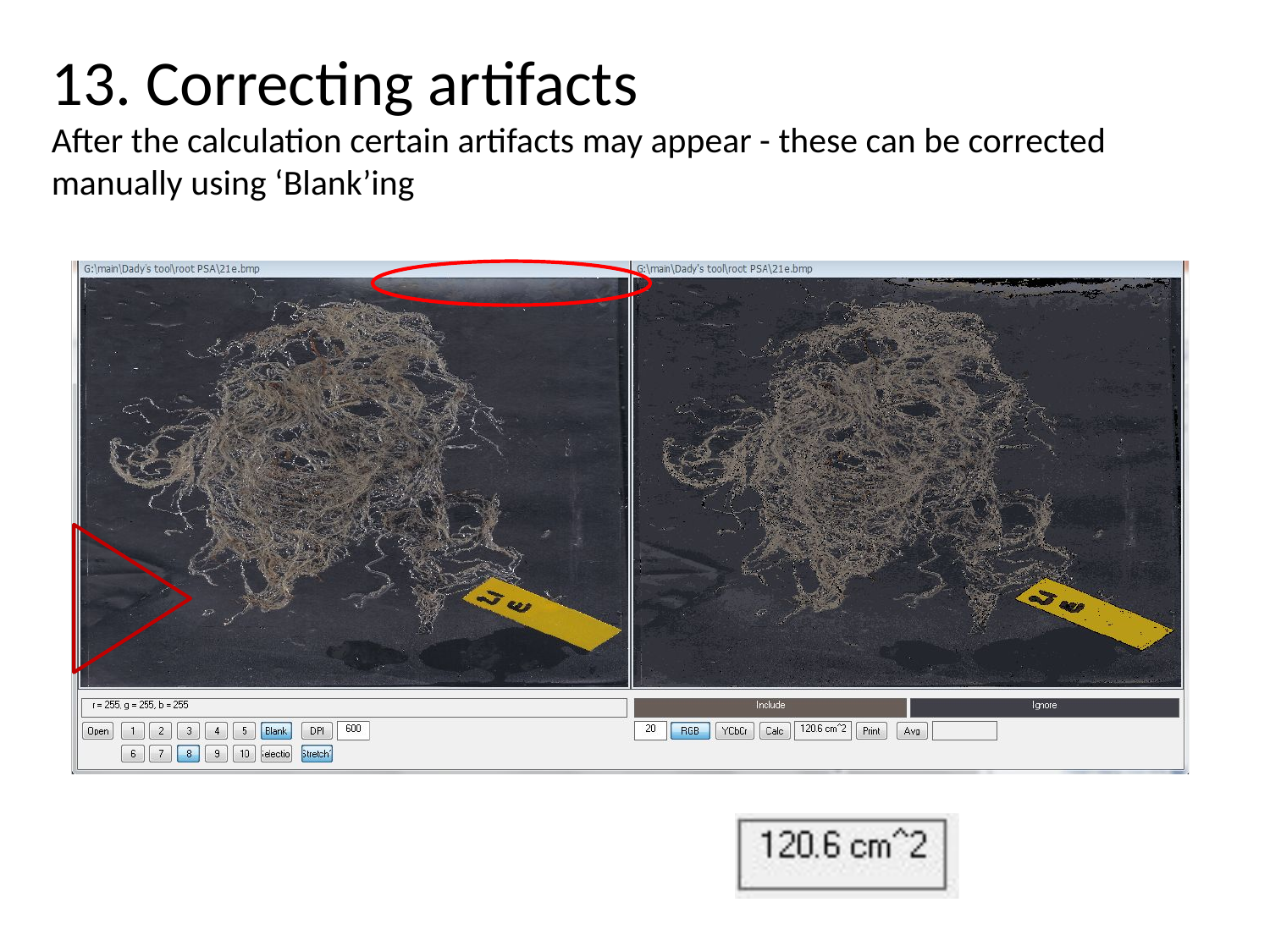

# 13. Correcting artifacts After the calculation certain artifacts may appear - these can be corrected manually using ‘Blank’ing

## Slide 19
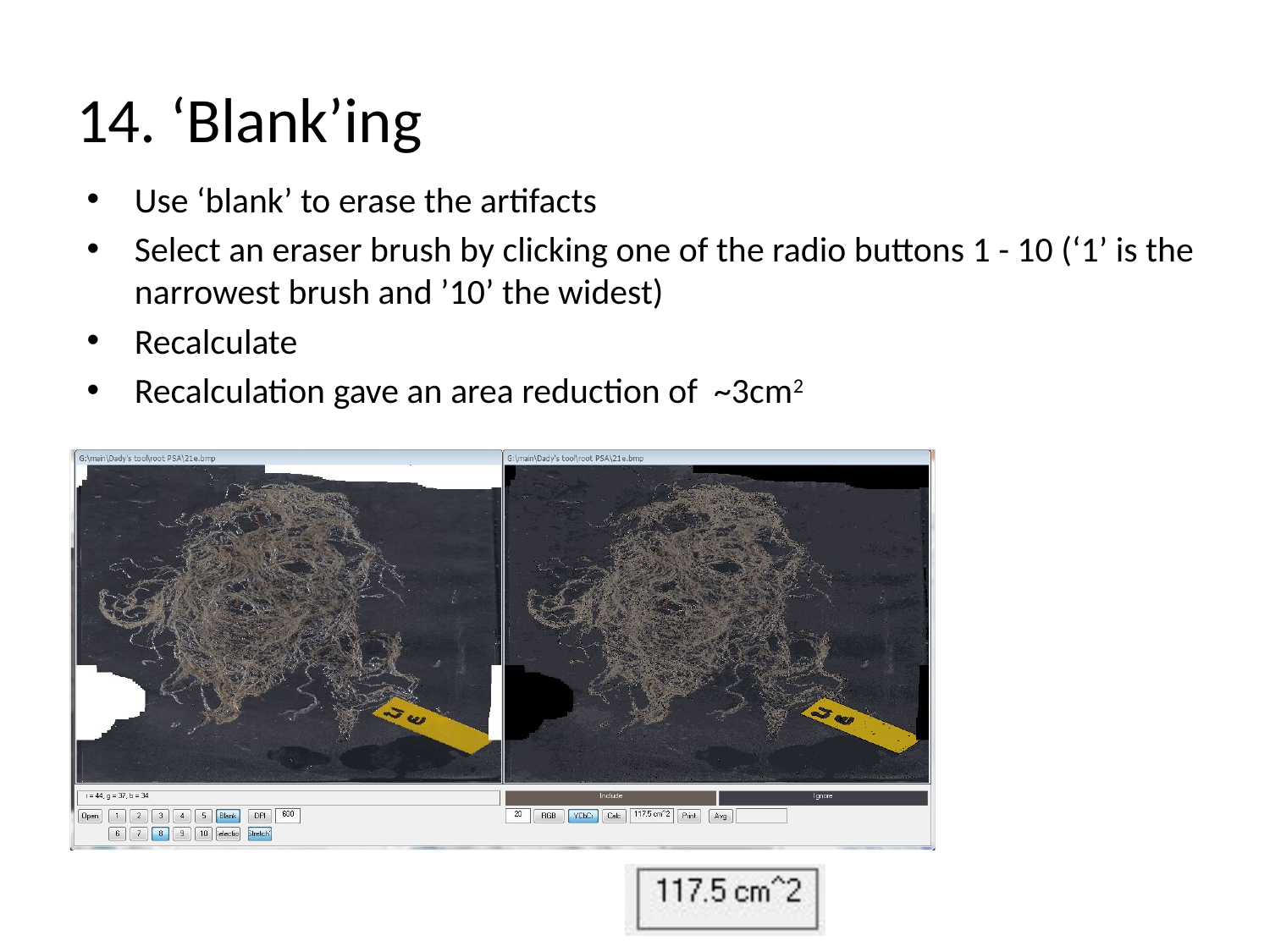

# 14. ‘Blank’ing
Use ‘blank’ to erase the artifacts
Select an eraser brush by clicking one of the radio buttons 1 - 10 (‘1’ is the narrowest brush and ’10’ the widest)
Recalculate
Recalculation gave an area reduction of ~3cm2
