# Supplementary figures and images for: CoverageTool: A semi-automated graphic software: applications for plant phenotyping
Source: Plant Methods. 2019 Aug 6;15:90. doi: 10.1186/s13007-019-0472-2 (PMC6683572; doi:10.1186/s13007-019-0472-2)

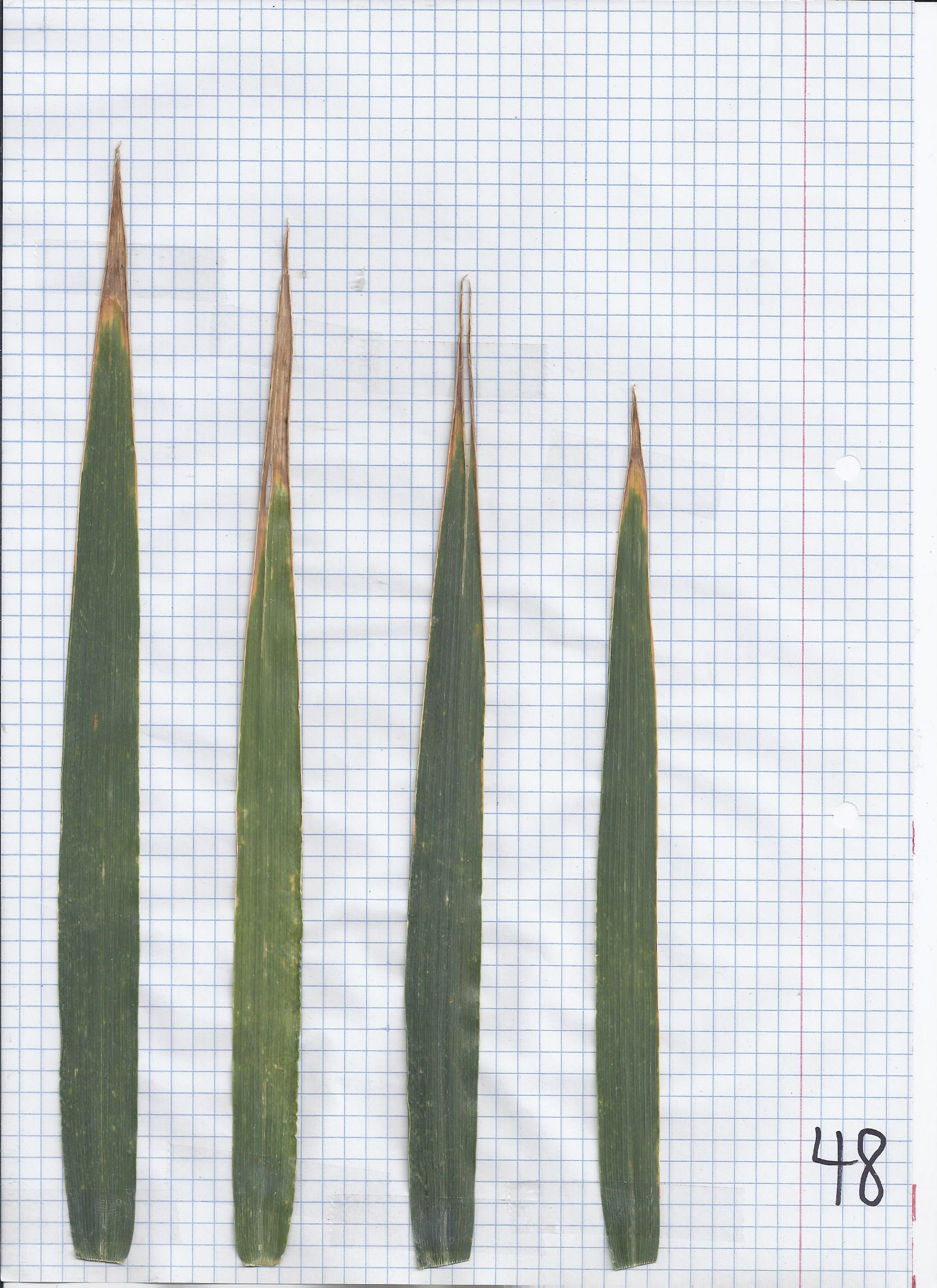

Supplement: Supplementary file 5 — Additional file 5. Wheat scanned leaf blades, 24-bit BMP file. [file 13007_2019_472_MOESM5_ESM.bmp]

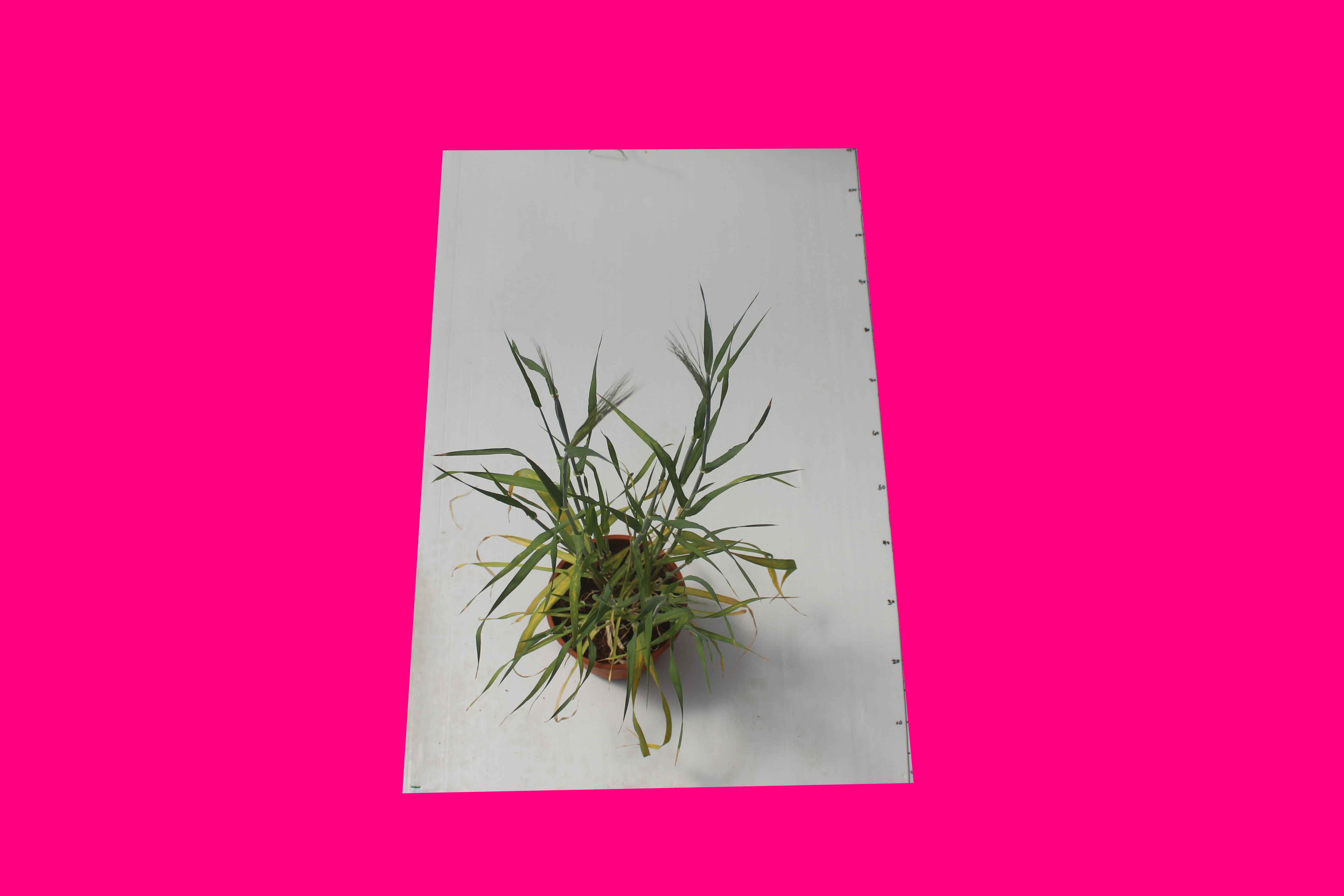

Supplement: Supplementary file 7 — Additional file 7. Barley shoot image, 24-bit BMP file. [file 13007_2019_472_MOESM7_ESM.bmp]

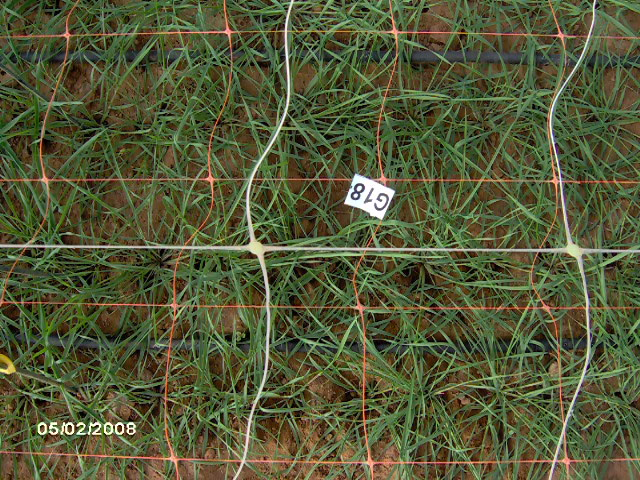

Supplement: Supplementary file 8 — Additional file 8. Wheat plot from above, early ground cover, 24-bit BMP file. [file 13007_2019_472_MOESM8_ESM.bmp]

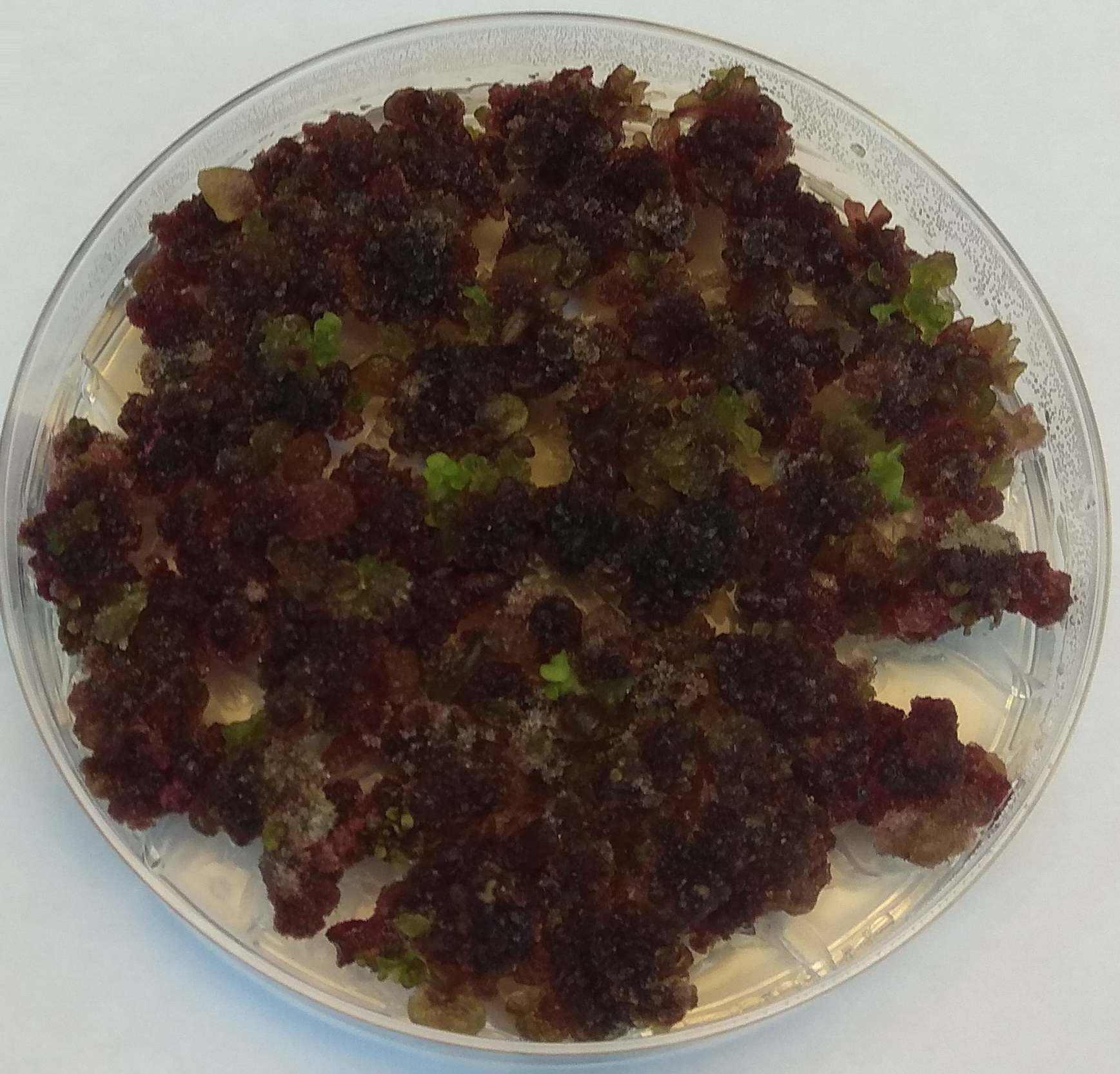

Supplement: Supplementary file 10 — Additional file 10. Nicotiana tabacum SR1 transformer with X11 calli, cropped, 24-bit BMP file. [file 13007_2019_472_MOESM10_ESM.bmp]

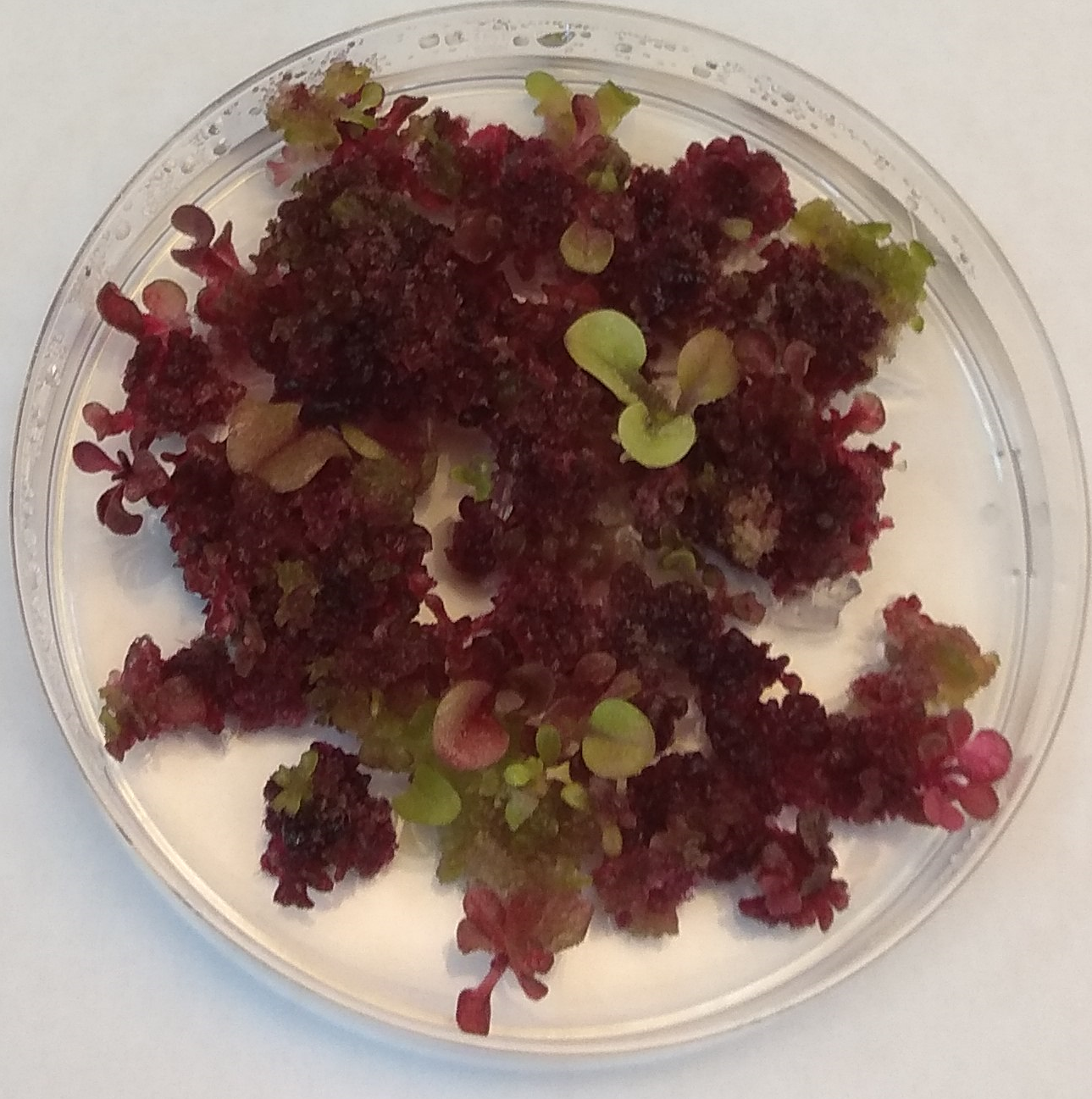

Supplement: Supplementary file 11 — Additional file 11. Nicotiana tabacum SNN transformer with X11calli cropped, 24-bit BMP file. [file 13007_2019_472_MOESM11_ESM.bmp]
