# Supplementary material for: CoverageTool: A semi-automated graphic software: applications for plant phenotyping
Source: Plant Methods. 2019 Aug 6;15:90. doi: 10.1186/s13007-019-0472-2 (PMC6683572; doi:10.1186/s13007-019-0472-2)
Supplement: Supplementary file 9 — Additional file 9. Transformation protocol and culture media. [file 13007_2019_472_MOESM9_ESM.docx]

**Supplementary 9**

**Basal media (pH to 5.8 with NaOH before adding the agar)**

**MS with vitamins (duchffa) = 4.41 g/L**

**Sucrose 2% = 20 g/L**

**Agarose (plant agar) 0.8% = 8 g/L**

**First callus induction medium:**

|  | **Final conc.** | **Stock solution** | **Amt. to add to 400 ml of basal media** |
| --- | --- | --- | --- |
| **Thidiazuron** | **0.4 mg/L** | **1 mg/mL** | **160µL** |
| **NAA** | **0.2 mg/L** | **1 mg/mL** | **80µL** |

**Second callus induction medium:**

|  | **Final conc.** | **Stock solution** | **Amt. to add to 400 ml of basal media** |
| --- | --- | --- | --- |
| **Thidiazuron** | **0.4 mg/L** | **1 mg/mL** | **160µL** |
| **NAA** | **0.2 mg/L** | **1 mg/mL** | **80µL** |
| **Kanamycin** | **100 mg/L** | **50 mg/mL** | **800 µL** |
| **Carbenicillin** | **120 mg/L** | **50mg/ml** | **960µL** |

**Regeneration medium**

|  | **Final conc.** | **Stock solution** | **Amt. to add to 400 ml of basal media** |
| --- | --- | --- | --- |
| **IAA** | **2mg/mL** | **0.5 mg/mL** | **800µL** |
| **Zeatin** | **2.5mg/mL** | **0.5mg/mL** | **1000µL** |
| **Kinetin** | **5mg/mL** | **0.5mg/mL** | **2000µL** |
| **Kanamycin** | **100 mg/L** | **50 mg/mL** | **800 µL** |
| **Carbenicillin** | **120mg/mL** | **50mg/mL** | **960µL** |

**Procedure**

*Always work in a flow hood, close to a flame

*Use forceps & scalpel sterilised with EtOH & flamed

*Seal plates with parafilm before removing from the flow hood Prepare the Agrobacterium:

♣ Inoculate 5 ml LB buffer with X11 transformed EHA105 strain in a 50 ml falcon tube using kanamycin (50mg/l final)

♣ Grow, shaking at 28ºC o/n to create a starter

♣ Add 1ml starter to 4ml fresh LB and kanamycin (50mg/l final)

♣ Grow, shaking at 28ºC o/n to create a starter

♣ Add 2ml starter to 25ml fresh LB and kanamycin (50mg/l final) and Acetosyringone (200µM)

♣ Grow, shaking at 28ºC until reach ~0.5 O.D

♣ Young leaves were excised using a sterilized forceps & place onto a sterile Petri dish 2 days before transformation procedure

♣ Pellet Agrobacteria at 4000rpm, 10min, and re-suspended with liquid MS supplemented with 20gr/l sucrose.

♣ The leaf segments are dipped into the Agrobacterium mix, incubated in the dark for 10min, and then soaked over a sterile paper an set on the 'First callus induction medium' for two days.

♣ Leaf segment were transferred onto 'Second callus induction medium' which contains the antibiotics selection for ~ 8 weeks, replacing fresh media every 2-3 weeks.

♣ Upon callus growth the were transfers to an 'Regeneration media' which contains the antibiotics selection
