# Supplementary material for: CoverageTool: A semi-automated graphic software: applications for plant phenotyping
Source: Plant Methods. 2019 Aug 6;15:90. doi: 10.1186/s13007-019-0472-2 (PMC6683572; doi:10.1186/s13007-019-0472-2)
Supplement: Supplementary file 12 — Additional file 12. Summary of the different case-studies: image type, scale, Supp. file, phenotyping target, parameter calculated, units, sample to ‘coverage’ and/or to ‘ignore’, tolerance, RGB/YCbCr metric, and references. [file 13007_2019_472_MOESM12_ESM.docx]

| **Study-case** | **1** | **2** | **3** | **4** | **5** |
| --- | --- | --- | --- | --- | --- |
| **Image type** | **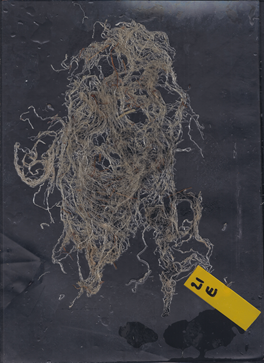** | **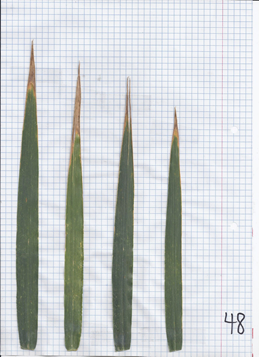** | **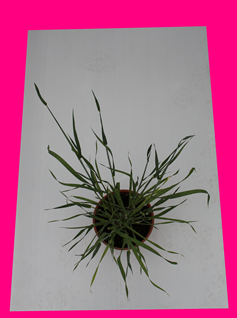** | **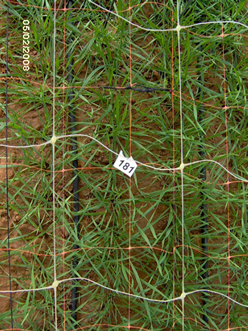** | **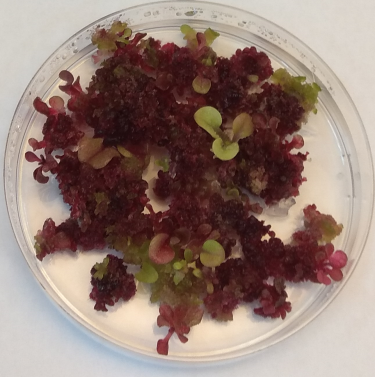** |
| **Scale** | Roots scanned over an A4 paper | Leaf blades scanned over an A4 | Aboveground plants in Pots | Aboveground field plot | Petri dish |
| **Supp. file** | 3 | 4 | 5 | 6 | 7,8 |
| **Phenotyping target** | Root | leaf | shoot | Canopy | Tissue culture |
| **Parameter Calculated** | Root projected surface area | Flag leaf blade projected surface area | Senescence  Area ratio | Early ground cover by canopy | Percentage of green regeneration vs. red in X11 transformed Tabaco |
| **units** | cm^2^ | cm^2^ | % | % | % |
| **Target** | Sample roots colors | Sample for leaf colors | a)'all': greens & yellow-brown  b)'yellow brown'  c)'yellow brown'/'all' | Sample canopy colors | a) 'all': reds & greens  b) 'greens'  c) 'greens'/'all' |
| **Sample to**  **'Ignore'** | Yes  Black/ gray background | No | Yes  Pink background cropping | Yes  White and orang netting and plot's numbering | No |
| **RGB/YCbCr** | RGB | YCbCr | YCbCr | RGB | YCbCr |
| **References** | [9] | [11] | [10] | [12] |  |

**Table S1.**
